# Prescribed burns drive lasting changes in soil nitrogen cycling and microbial function

**DOI:** 10.1128/msystems.00852-26

**Published:** 2026-09-21

**Authors:** Alaina O. Benot, Gray Waldschmidt, Samuel C. Gilvarg, Eva O. L. Legge, Isaac J. Okyere, Chanistha Tiyapun, Sarah K. Lucas, Andrew L. Vander Yacht, Jennifer L. Goff

**Affiliations:** 1 Department of Environmental Biology, SUNY College of Environmental Science and Forestry 14797, Syracuse, New York, USA; 2 Department of Sustainable Resources Management, SUNY College of Environmental Science and Forestry 467851, Syracuse, New York, USA; 3 Department of Biology, Syracuse University 171413, Syracuse, New York, USA; 4 Department of Chemistry, SUNY College of Environmental Science and Forestry 539343, Syracuse, New York, USA; Stellenbosch University, Stellenbosch, South Africa

**Keywords:** Albany Pine Bush Preserve, nitrogen, denitrification, nutrient cycling, fire ecology, metagenomes, soil, forest, pine barrens

## Abstract

Fire is a major pulse disturbance to soil microbial communities, with broad implications for nutrient cycling; however, regular burning is also a natural and often-essential process maintaining biodiversity in unique and imperiled fire-dependent ecosystems. Prescribed fire is widely used to promote this biodiversity and simultaneously reduce wildfire risk. Although such repeated burning is known to alter surface biodiversity, belowground soil geochemistry, and soil microbial community structure, the functional consequences (i.e*.*, the metabolic capabilities that underlie the ecosystem services soil microorganisms provide) remain underexplored. Here, we examined the effects of 30 years of repeated prescribed fire at the Albany Pine Bush—a fire-dependent, inland pitch pine barren ecosystem of the northeastern United States. Compared with the control stands, we observed that this long-term fire management has led to substantial depletion of inorganic soil nitrogen, specifically nitrate. We found no meaningful differences in the higher-level taxonomic composition of soil prokaryotic or fungal communities; however, analysis of metagenome-assembled genomes assembled from these soils revealed several differentially abundant populations. Furthermore, our metagenomic analysis revealed significant changes in the nitrogen-cycling functional potential, specifically decreased dissimilatory nitrate reduction and denitrification potential in repeatedly burned soils. These functional shifts have important implications for both nutrient cycling and emissions of trace nitrogen gases from these soils. Our results suggest that functionally meaningful changes in the soil microbiome can persist between burn events, even when higher-order community membership appears stable. This may imply that repeated fire can deplete reactive nitrogen emissions from soils by lowering the functional capacity of nitrogen-reducing microbes.

**IMPORTANCE:**

Prescribed fire is widely used by land managers to reduce wildfire risk and promote biodiversity. While the effects of fire on aboveground processes are well understood, much less is known about how repeated burning influences soil biological properties—including the functional role that soil microorganisms play in nutrient cycling and greenhouse gas production. We addressed this gap by studying soils from the Albany Pine Bush, a rare and endangered ecosystem that has experienced regular prescribed fires for 30 years. Long-term fire management significantly altered soil chemistry, specifically lowering the amount of nitrogen in the soil. In addition, we found that fire management decreased the genetic potential of the soil microbial community to produce nitrogen oxides—potent contributors to climate change. Thus, prescribed fire’s contribution to greenhouse gas emissions may involve a complex relationship between direct fire-driven emissions, increased fire resilience of promoted vegetation, and—as suggested by our results—the reduced ability of soil microbes to produce greenhouse gases.

## INTRODUCTION

Over millennia, many biodiverse ecosystems have evolved with fire and require regular burning to persist (1–3). To maintain these ecosystems while reducing wildfire risks, land managers can actively apply fire to landscapes through prescribed burning (4). As a result, prescribed fire is a widely used land management tool that generates $3.73 in societal benefits for every dollar invested (5). Repeated prescribed burns significantly alter aboveground ecosystem properties and biodiversity (6–9) as well as belowground soil geochemistry (10, 11)—both of which can strongly shape the structure and function of soil microbial communities.

Our current understanding of how prescribed fire shapes soil microbial community structure and function is derived, in part, from studies of immediate post-burn responses in systems that have not yet transitioned to the state maintained by long-term exposure to historical patterns in fire frequency, intensity, severity, seasonality, and spatial extent (i.e., ecosystems exposed to prescribed fire following extended fire suppression). This research suggests that compositional shifts following a single, initial prescribed fire are generally modest and transient, often dissipating within weeks to months (12–16). Far less is known about functional changes in microbial communities following prescribed burning, but limited evidence (two studies) indicates that shifts in carbon and nitrogen cycling are also transient. A single prescribed burn in a tallgrass prairie in the northwestern United States enriched bacterial metagenome-assembled genomes (MAGs) with increased aromatic carbon degradation and reduced polysaccharide degradation potential. However, these functional gene shifts peaked within 1 week and returned to baseline by 5 months (16). Similarly, in a southeastern U.S. mixed hardwood forest, nitrification-associated prokaryotes were enriched immediately post-burn, depleted by 3 months, and returned to baseline after 17 months (12).

Systems restored and maintained by regularly applied prescribed fire differ fundamentally from a single fire after prolonged suppression, and such differences appear to result in more substantial and longer-lasting effects on soil microbial communities. For example, in longleaf pine forests in the southeastern United States, 30+ years of prescribed burning (every 2–4 years, depending on the site) altered both fungal and bacterial community structures in topsoils (17–19). Similar patterns have been observed in Australian wet sclerophyll forests (20, 21), tallgrass prairies in the midwestern United States (22), a temperate Australian grassland (23), a semi-arid savanna in South Africa (24), and a peatland in the United Kingdom (25). In combination, these prior studies suggest that repeated application of prescribed fire may drive more pronounced community restructuring than a single prescribed fire. However, far less is known about how these fire regime shifts influence the soil microbial community’s function.

Soil microorganisms are predicted to play a central role in post-fire ecosystem recovery by regulating nutrient cycling (26), soil structure (27), and plant-microbe interactions (28) that influence vegetation regeneration and productivity (29–31). Because vegetation affects fuel accumulation and future fire behavior, fire-induced changes in microbial communities may contribute to plant-soil-fire feedbacks that shape long-term ecosystem trajectories (32, 33). Soil microbial communities also mediate the production and consumption of greenhouse gases, linking post-fire microbial activity to ecosystem carbon and nitrogen budgets (34). While prescribed fire generates an immediate pulse of greenhouse gas emissions during combustion (35), its long-term climatic effects will depend on how repeated burning alters microbial processes between fire events (36). Despite the importance of these microbial processes, relatively little is known about how repeated prescribed fire influences the functional potential of the soil microbial community. Here, we examine how repeated application of prescribed fire affects the soil microbial community at the Albany Pine Bush Preserve (APBP), an inland pine barrens ecosystem with a contemporary prescribed-burn history dating back more than 30 years (37, 38). Using metagenomic sequencing, we characterized both the taxonomic composition and functional potential of soil microbial communities across contrasting fire-return intervals. This approach enabled us to evaluate how repeated prescribed fire shapes the microbial community and alters the genetic capacity for key biogeochemical processes.

## MATERIALS AND METHODS

### Sampling location and methods

The APBP (Albany, NY, USA, 42.71°N, 73.86°W) is a fire-dependent inland pine barrens ecosystem with a contemporary prescribed-burn history dating back more than 30 years (37, 38). Historically, indigenous communities used fire for millennia to foster healthy and productive ecosystems (37, 39–42), and the earliest evidence of fire within the APBP dates to approximately 6,400 years ago (43). However, since the early 1900s, urbanization and a strict policy of fire suppression led to the near-complete exclusion of fire from the barrens (44). Since 1991, the APBP has enacted a forest management plan that has included the reintroduction of fire—the ecological effects of which are well documented aboveground (6, 7, 43, 45–51). Details on the site’s botany and geography can be found in the Supplemental information.

**Fig 1:**
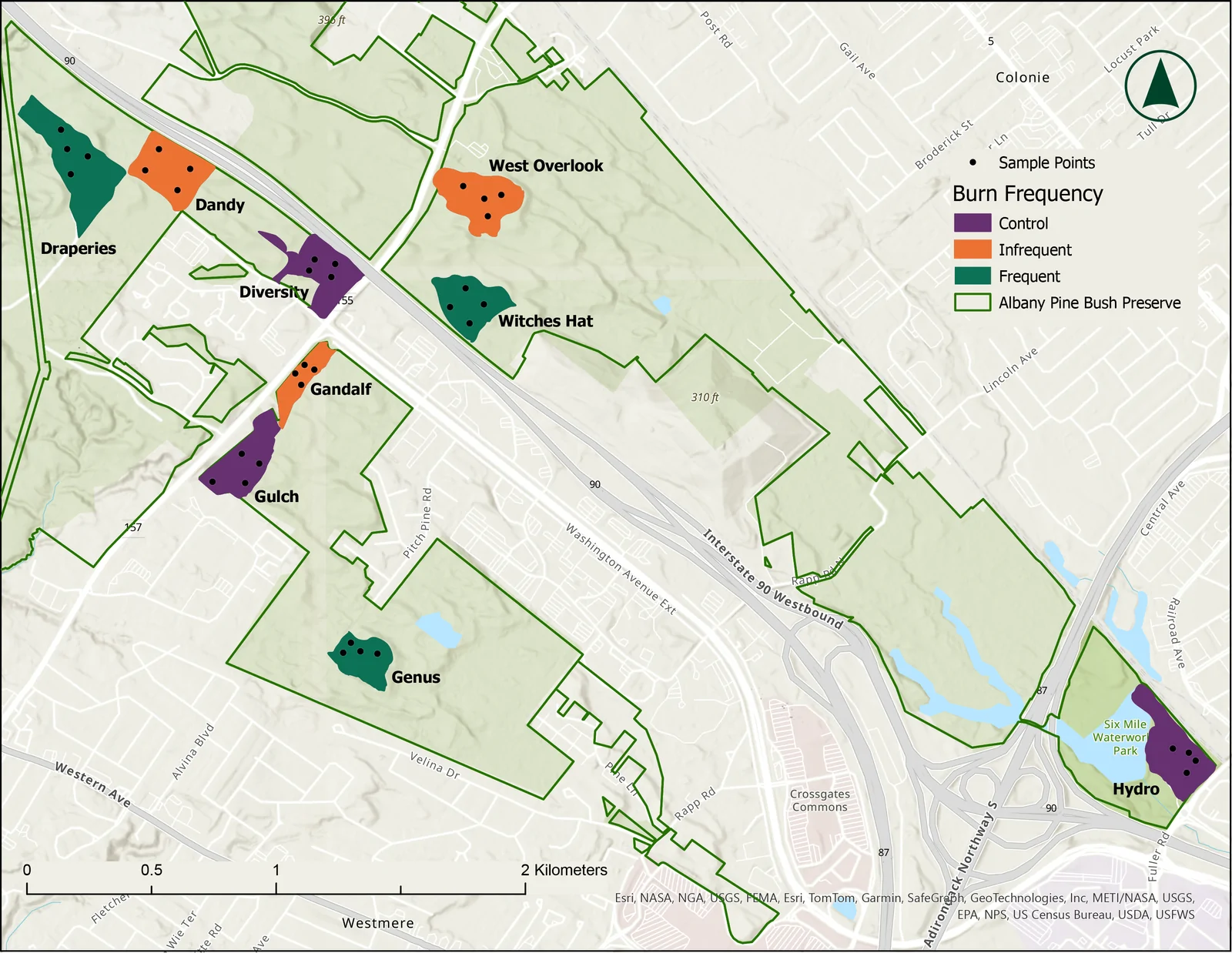
Sampling plots within the Albany Pine Bush (outlined in dark green). Control (purple) stands have not experienced fire since at least 1991. Frequently (green) burned stands have an average burn interval of ~7 years, and infrequently (orange) burned stands have an average fire return rate of ~16.5 years. Four sampling plots within each stand are denoted by black dots, identified by latitude and longitude coordinates. Samples collected within a single stand were homogenized at each depth to create a single, representative sample for that stand.

On 8 November 2024, we sampled soils from nine stands at the APBP. Stands were intentionally selected to encompass the variation in fire frequency that has occurred over the past 30 years of active management, with those that have been burned ≥4 times (average fire return interval = 6.5–8.25 years) labeled as “frequent,” those that experienced >1 but ≤3 fires (average fire return interval = 16.5 years) labeled as “infrequent,” and those that have no record of fire over this period labeled as “control” (Fig. 1; Fig. S1, Tables S1 and S2). This categorization captured the difference between stands that have (i) recently burned at a frequency aligned with historic fire return intervals (“frequent”), (ii) recently burned, but not as often as they would have historically (“infrequent”), and (iii) continued to experience the fire suppression that characterizes the broader region (“control”) (7). The persistence of pitch pine barrens largely depends on the restoration of historical fire regimes but involves intermediate steps. Thus, our work across this gradient of restoration states can directly inform how prescribed fire alters fire, soil, and microbial community feedbacks over time. Sampling this gradient is also important, as prior work has suggested that different fire return interval lengths differentially impact microbial community responses to fire (18, 20). Although fire behavior characteristics for individually applied burns were not available, each “frequent” and “infrequent” stand was burned according to a similar prescription (i.e., weather and fuel conditions); hence, fire behavior likely did not differ drastically between stands.

As fires do not naturally burn evenly across stands, we established four randomly selected plots within nine 5- to 20-hectare stands at the APBP to capture this heterogeneity. For all selected stands, the most recent fire was at least a full year before our observations. Within each stand, sampling plots were randomly located a minimum of 35 m from the edges of the preserve to minimize edge effects (Fig. 1). At each plot, we extracted two soil cores—one for each of two soil depths: 0–15 and 15–30 cm (*n* = 72). These samples were then homogenized by soil depth within stands to yield three samples per fire treatment (“control,” “infrequent,” and “frequent”) at two soil depths (total *n* = 18) and stored at −80°C until analysis (Table S3).

### Soil geochemical measurements

Geochemical analyses were performed at the University of Maine Analytical Laboratory and Maine Soil Testing Service. Metrics analyzed were pH, percent organic matter, percent total nitrogen (%TN), percent total carbon (%TC), exchangeable acidity, effective cation exchange capacity (ECEC), ammonium chloride-extractable-calcium (Ca), potassium (K), magnesium (Mg), phosphorus (P), aluminum (Al), iron (Fe), manganese (Mn), sodium (Na), zinc (Zn), nitrate (NO_3_-N), and ammonium (NH_4_-N). First, soils were spread in a 2-mm layer for air-drying. Soil pH was then measured in distilled water (1:1, soil:water). Organic matter was determined by loss on ignition (LOI) at 550°C for 5 h. %TN and %TC were measured by combustion analysis at 1,350°C. NO_3_-N and NH_4_-N were first extracted in potassium chloride (1 N, 1:10, soil:solution) and then measured colorimetrically by flow injection analysis using the cadmium reduction/sulfanilamide method for NO_3_-N and hypochlorite/salicylate method for NH_4_-N. All other nutrients were extracted in ammonium chloride (1 N NH_4_Cl at a 1:20, soil:solution) and measured by inductively coupled plasma optical emission spectroscopy (ICP-OES). Exchangeable acidity was extracted in potassium chloride (1 N KCl at a 2.5:50, soil:solution) and measured by NaOH titration. ECEC was calculated by the summation of exchangeable base cations plus exchangeable acidity.

### Metagenomic sequencing and analysis

The short-read metagenomic sequencing methods used are described in Benot et al. (52). Briefly, short-read sequencing was performed on an Element AVITI using Cloudbreak Freestyle chemistry—generating approximately 36–40 Gb of data per sample of 150 bp, paired-end reads. Trimmomatic v0.39 (53) was used for read trimming and adaptor removal, followed by quality assessment using FastQC (54). Taxonomic profiles for the quality-controlled metagenomic reads were generated using Kaiju (v1.9.0) (55) (using the NCBI BLAST nr and NCBI RefSeq fungi databases), run on the U.S. Department of Energy’s Knowledge Base (KBase) platform (56). Functional profiles of nitrogen-cycling genes were generated using the Fama pipeline, as previously described (57–59). Briefly, quality-controlled reads were used as input to the Fama pipeline (60), as detailed in the Supplemental information, which was implemented in KBase. The genes within each pathway are defined here. The Supplemental information file also contains a table describing the genes contained within each pathway grouping (Table S4) and an accompanying pathway map (Fig. S2).

### Metagenome assembly, binning, and analysis

Metagenomic assembly, binning, and annotation were performed within KBase as described in a previous study by Benot et al. (52). Briefly, reads were assembled using MetaSPAdes (v3.15.3) (61). Assembled contigs were binned using MetaBAT2 (v1.7) (62), CONCOCT (v1.1) (63), and MaxBin2 (v2.2.4) (64). DAS Tool (v1.1.2) (65) was used to dereplicate and refine the resulting bins from each sample. All MAGs were then dereplicated at 95% ANI using FastANI (v. 0.1.3) (66). The resulting metadata for all MAGs are reported in Benot et al. (52). The assembled MAGs are publicly available on figshare at https://figshare.com/articles/dataset/Archive_of_MAGs_assembled_from_the_Albany_Pine_Bush_Preserve_soil_metagenomes_/29424812?file=55727801. MAGs were annotated using DRAM (v0.1.2) in KBase (67). MAG abundances were calculated using CoverM (v0.7.0) to align the reads to the dereplicated MAGs and calculate coverage and relative abundances (68). A phylogenetic tree containing all the MAGs was constructed using the KBase application Insert Set of Genomes Into SpeciesTree (v2.2.0), which generates a multiple-sequence alignment of 49 universal genes. Alignments are then trimmed using GBLOCKS (69) and concatenated, and a phylogenetic tree is reconstructed using FastTree2 (70). The R package “treeio” was used for visualizations (71).

### Statistical methods

Comparisons of soil geochemistry between treatments were performed using a two-way analysis of variance (ANOVA) with a *post hoc* Tukey’s honestly significant difference (HSD) test conducted using the aov and TukeyHSD functions in the base R “stats” package (v4.3.1). A principal coordinate analysis (PCoA) was conducted using the base R “stats” package prcomp function on a Bray-Curtis distance matrix.

Comparisons of community composition between treatments were performed using a permutational multivariate analysis of variance (PERMANOVA) conducted in R using the “vegan” package (v2.7-1) adonis2 function (Bray-Curtis and Jaccard distance matrices with permutations = 999). Differential abundance of MAGs across the treatments was determined using “DESeq2” (v1.42.1) in R (72). A model comparing the three treatments was fitted with soil depth included as a blocking factor. Pairwise contrasts comparing each burned treatment to the unburned control were extracted using the Wald test. Adjusted *P*-values were calculated using the Benjamini-Hochberg procedure using the base R “stats” package p.adjust function. MAGs with a false-discovery rate <0.05 and a |log_2_ fold change| >1 were considered differentially abundant (72).

Comparisons of N-cycling gene composition between treatments were performed using PERMANOVA, as described above, with a Bray-Curtis distance matrix. The beta-diversity of the N-cycling genes was visualized using the “vegan” package metaMDS for non-metric multidimensional scaling (Bray-Curtis distance matrix) (73). To identify correlations between N-cycling gene abundances and soil nitrate concentrations, we performed Spearman correlation analyses using the base R “stats” package cor function, followed by a Benjamini-Hochberg correction, as described above. Comparisons of specific genes and pathways between treatments were performed using a one-way ANOVA with a *post hoc* Tukey’s HSD test conducted using the aov and TukeyHSD functions in the base R “stats” package (v4.3.1), followed by a Benjamini-Hochberg correction, as described above.

The R packages “ggplot2” (v4.0.3) (74) and “Complex Heatmap” (v2.24.1) (75) were used for various visualizations.

## RESULTS AND DISCUSSION

### Prescribed fire alters soil nitrogen chemistry

**Fig 2:**
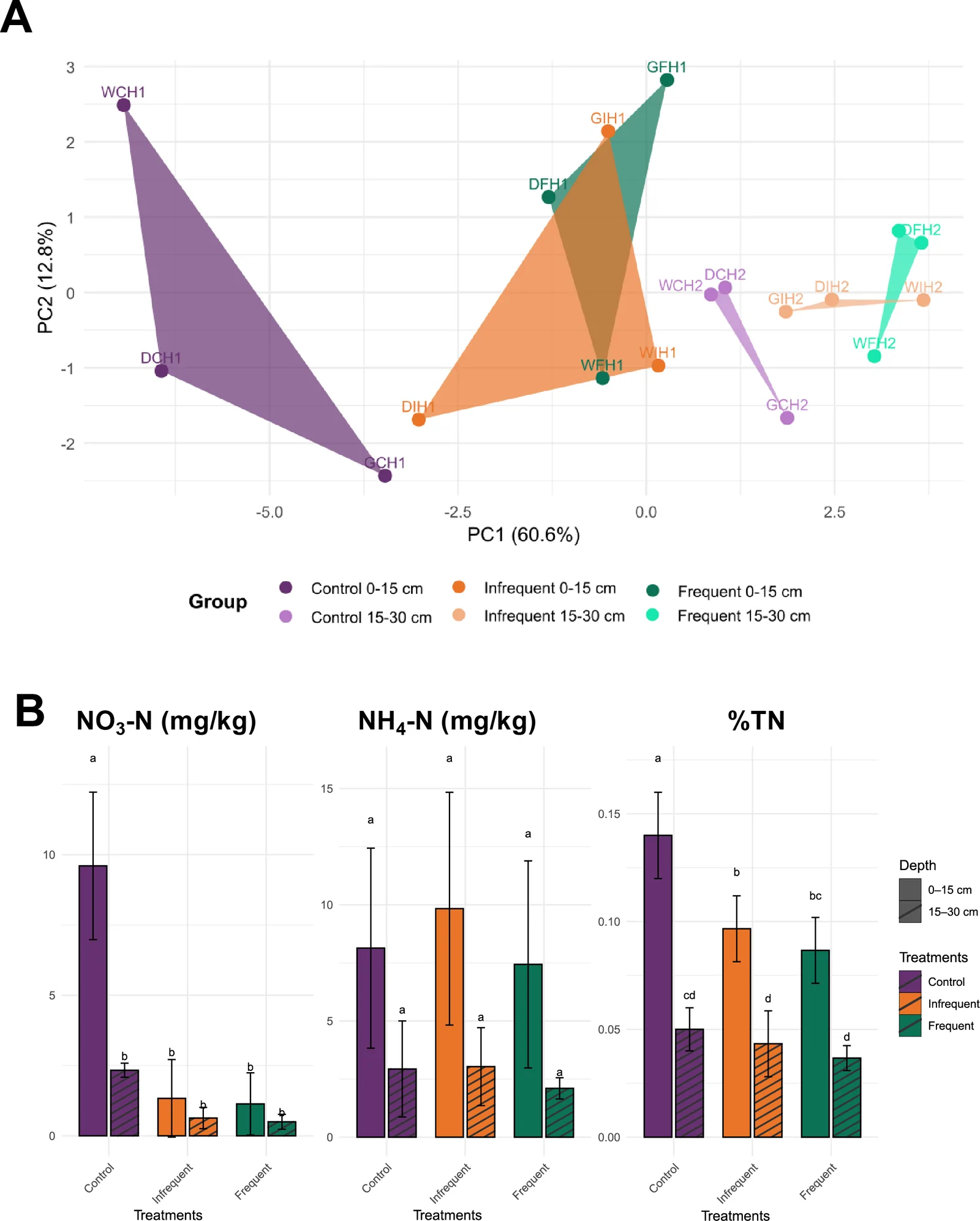
(**A**) PCoA plot (Bray-Curtis dissimilatory) of the stand soil geochemistry using homogenized soil samples (per stand: one for the 1- to 15-cm depth and one for the 15- to 30-cm depth). Colored polygons are used to group samples by treatment and depth. Sample names are labeled, with descriptions in Table S3. (**B**) Averaged concentrations of nitrate (NO_3_^−^-N, mg/kg), ammonium (NH_4_^+^-N, mg/kg), and percent total nitrogen (%TN) across all fire treatments and soil depths. Solid and striped columns represent 0- to 15-cm and 15- to 30-cm depths, respectively. Two-way analysis of variance (ANOVA) with *post hoc* Tukey’s HSD test was performed. Letters above bars indicate significant differences (*P* < 0.05) among treatments across the two depths; bars sharing the same letter are not significantly different. Error bars represent standard deviation (±SD).

Repeated prescribed fire significantly influenced soil chemistry at both shallow (0-15 cm) and deeper (15-30 cm) depths (Tables S5 and S6, Fig. S3 to S7). PCoA of the geochemical data revealed separation of stands by both fire management history and depth (Fig. 2A).

Observed differences across the fire treatments in the soil geochemical data included pH, Fe concentrations, and ECEC (*P* < 0.05, two-way ANOVA with Tukey’s HSD). Soil pH increased by 10% at the 0- to 15-cm depth in the burned stands relative to the controls (Fig. S7). This increase in surface soil pH is commonly observed in post-fire systems and is typically attributed to alkaline ash deposition and the combustion of organic acids (76). Although often considered short-lived, moderate pH increases have been reported to persist in some systems managed with repeated prescribed burns (20, 77–79). Ammonium chloride-extractable Fe decreased 2-fold between control and frequently burned stands at the 0- to 15-cm depth (Fig. S4). Similar decreases in extractable Fe have been reported previously post-fire (80–83), which have been attributed to fire-associated leaching, erosion, and precipitation into an insoluble form (81). ECEC also decreased at the 0- to 15-cm depth in the infrequently burned stands relative to the control stands (Fig. S7). While ECEC also trended in the same direction in the frequently burned stands, it did not achieve our threshold of significance. Prior work has shown that soil cation exchange capacity (CEC) at the surface typically decreases due to the combustion of soil organic matter, although thermal conversion of soil minerals also contributes to this decrease (84–87).

At both depths, we also observed significant shifts in soil nitrogen (N) chemistry. At 0–15 cm, %TN decreased ~1.5-fold in the burned stands compared to controls (Fig. 2B, two-way ANOVA with Tukey’s HSD, *P* < 0.05 for both treatments). Although similar trends were observed at the 15- to 30-cm depth in the burned stands, these decreases were not statistically significant. During prescribed burns, N losses can occur when organic N compounds thermally decompose at temperatures >200°C (88) and through increased rates of erosion and leaching due to hydrogeologic changes in the soil post-fire (89, 90). However, the literature on the effects of repeated prescribed fire on %TN is mixed, with studies reporting increases, decreases, or no significant changes (11). These variable responses have been attributed to differences in the plant composition, burn histories, and initial soil characteristics of the different sites (79, 91, 92).

As %TN integrates multiple inputs and losses, we also examined inorganic N species to assess how fire influences N bioavailability and cycling. No differences in NH_4_-N were observed between treatments (Fig. 2B; two-way ANOVA, *P >* 0.05). However, NO_3_-N declined substantially with burning at both depths, with 9-fold (frequent) and 5-fold (infrequent) reductions in the surface soils and similarly substantial declines at the 15- to 30-cm depth (5-fold and 4-fold, respectively) (Fig. 2B; two-way ANOVA with Tukey’s HSD, *P <* 0.05). Post-fire dynamics of NH_4_-N and NO_3_-N are complex (11), and our single time point limits our ability to resolve these temporal patterns. However, prior work provides general insight. Immediately post-burn, soils often exhibit a pulse of NH_4_-N (93–95), attributable to volatilization of organic N and subsequent NH_4_-N deposition (94, 95). The NH_4_-N then declines over time (typically 2–3 years) toward baseline levels (94, 95), as it serves as a substrate for prokaryotic nitrification (NH4^+^ → NO_2_^−^ → NO_3_^−^). During this period, an NO_3_-N pulse is often observed within 12 months post-fire, followed by a similar return to baseline within the subsequent 12 months (94).

In the APBP, however, we observed a significant depletion of NO_3_-N between prescribed burns at both 0- to 15-cm and 15- to 30-cm depths (Fig. 2B). This is consistent with an earlier meta-analysis finding that prescribed fire produces a smaller post-fire pulse of NH_4_-N and NO_3_-N than wildfire (94). As a result, substrate availability for nitrification is reduced, potentially contributing to NO_3_-N depletion (67–69, 96). We observed that NH_4_-N concentrations in the APBP burned stands were unchanged relative to the control stands. This finding is difficult to interpret on its own, as most of our sites fall within the typical window (2–3 years post-fire) during which NH_4_-N concentrations would be expected to return to their baseline values; however, even our most recently burned sites (1–2 years post-fire) showed NH_4_-N values similar to the control, suggesting that little additional substrate was generated post-fire for conversion to NO_3_-N. Fire-driven changes in soil hydrology (10) may further drive NO_3_-N loss through increased leaching and erosion (97). These potential pathways for NO_3_-N loss should be examined directly in future temporal studies.

Finally, within the soil profile, we expected fire to exert immediate and direct effects at the surface—with indirect effects extending belowground due to altered forest vegetation and microclimates (98), changes in nutrient inputs (97, 99), and shifts in the soil physical structure (76, 97, 100). However, prior studies of fire effects on soil chemistry and microbiota rarely capture depths greater than 15 cm below the surface (11, 101–105). Our study addresses this gap by capturing both shallower (0–15 cm) and deeper (15–30 cm) soil cores, allowing us to examine these more indirect effects, and indeed, we observed significant shifts in the soil chemistry at these deeper depths, emphasizing the value of extending cores deeper below the surface.

### Prescribed fire impacts microbial populations only at the genome scale

Next, we used read-based metagenomic profiling to evaluate whether these fire-driven changes in soil chemistry were also associated with altered microbial community composition. Across all soils, the most prevalent prokaryotic phyla were *Proteobacteria*, followed by *Acidobacteria* and *Actinobacteria* (Fig. S8; Tables S7 to S11). Soil depth significantly structured prokaryotic (*Bacteria* and *Archaea*) community composition from the phylum to genus levels (Fig. S8 to S12; PERMANOVA, *P* < 0.05; PERMANOVA results: Table S12), whereas fire treatment as well as its interaction with depth had no significant effect on microbial community composition (*P* > 0.05). The results were consistent across Bray-Curtis and Jaccard dissimilarity metrics. We also considered the fungal community composition, and the most prevalent fungal phyla were the *Ascomycota, Basidiomycota*, and *Mucoromycota* (Fig. S13 to S17; Tables S13 to S17). Like the prokaryotes, soil depth also structured fungal community composition at the family (PERMANOVA, Bray-Curtis: *R*^2^ = 0.1, *P* = 0.03; Jaccard: *R*^2^ = 0.1, *P* = 0.029) and genus levels (PERMANOVA, Bray-Curtis: *R*^2^ = 0.1, *P* = 0.025; Jaccard: *R*^2^ = 0.1, *P* = 0.021). Fire treatment, as well as its interaction with depth, had no effect (*P* > 0.05) at any taxonomic level (phylum down to genus) examined (all results: Table S18).

The impacts of repeated prescribed fire on soil microbial communities have been investigated in a limited number of systems, and our findings contrast with many of these studies. Significant fire-driven shifts in bacterial and/or fungal communities have been reported in an Australian wet sclerophyll forest (2- to 4-year fire return intervals) (20) (21), a southeastern U.S. longleaf pine forest (1- to 4-year fire return intervals) (17), a midwestern U.S. tallgrass prairie (annual burns) (22), a South African semi-arid savanna (1- to 6-year fire return interval) (24), and a U.K. peatland (10- to 20-year fire return intervals) (25). However, except for the U.K. study, these studies all involved shorter fire return intervals than those we explored at the APBP (~7 years in frequently burned and 16.5 years in infrequently burned stands). Prior studies have suggested that the extent to which prescribed fire restructures microbial communities may depend on the fire return interval, with shorter return intervals introducing more frequent perturbations. In the Australian wet sclerophyll forest, a 2-year return interval increased bacterial alpha-diversity more than a 4-year interval (20). Similarly, a 3-year return interval in a southeastern U.S. loblolly pine forest significantly shifted soil fungal communities, whereas a 6-year interval produced a composition similar to unburned controls (18).

We also considered that time since the last fire, rather than fire return interval, might differentially impact the microbial community structure. All our stands were last burned 1–5 years prior to sampling (17, 20, 22, 24, 25). Likewise, all the plots in the above-described studies were burned 1–6 years prior to sampling. A caveat to this statistic, however, is that—except for the U.K. peatland study—time since fire was also equivalent to the fire return interval in each system, making the two variables difficult to disentangle. Nonetheless, this comparison of our work with the broader literature leads us to speculate that fire return interval, rather than time since fire, has a greater impact on microbial community structure. Indeed, an earlier study in Australian grasslands found that return interval (i.e., disturbance frequency), rather than time since fire (i.e., time since disturbance), had a greater effect on soil community diversity and composition (23).

While our taxonomic profiles revealed no compositional differences between groups, this does not preclude divergence at the gene and genome scale. We therefore turned to genome-resolved metagenomics to characterize the site’s genomic content. We previously constructed MAGs from the APBP metagenomes and dereplicated the set (≥95% ANI) to obtain 94 species-level representatives (52) (Fig. S18; Table S19). Seven MAGs were differentially abundant under frequent burning relative to controls (Tables S20 to S22). Four of these MAGs were more abundant in frequently burned stands: a *Solirubrobacteraceae* (Bin.007.GFH2), a *Polyangiaceae* (Bin.013.GFH2), and a *Chthoniobacterales* (Bin.009.GFH2) species. Notably, both *Solirubrobacteraceae* and *Chthoniobacterales* have been previously identified in post-fire soils—attributed to their genetic potential for pyrogenic organic matter (PyOM) degradation (104). MAGs more abundant in controls included two *Dormibacteria* species (Bin.016.WIH1 and Bin.008.WIH1), a *Burkholderiales* species (Bin.001.DCH2), and a *Nitrosopumilaceae* species (Bin.008.DCH2). *Nitrosopumilaceae* are ammonia-oxidizing Archaea (AOA) that contribute to nitrification—the primary biological pathway supplying NO_3_-N to soils (106, 107). This suggests that fire-associated changes in some nitrifier populations could be one driver of the diminished soil NO_3_-N. We note, though, that despite sequencing to a depth of ~36–40 Gb/sample, these MAGs accounted for only ~2.5%–7.5% of total reads per sample (Table S23). While this level of representation is not unusual for complex soil metagenomes, it nonetheless reflects a highly incomplete reconstruction of the site’s total microbial diversity (108).

### Prescribed fire decreases nitrate reduction and denitrification potential

**Fig 3:**
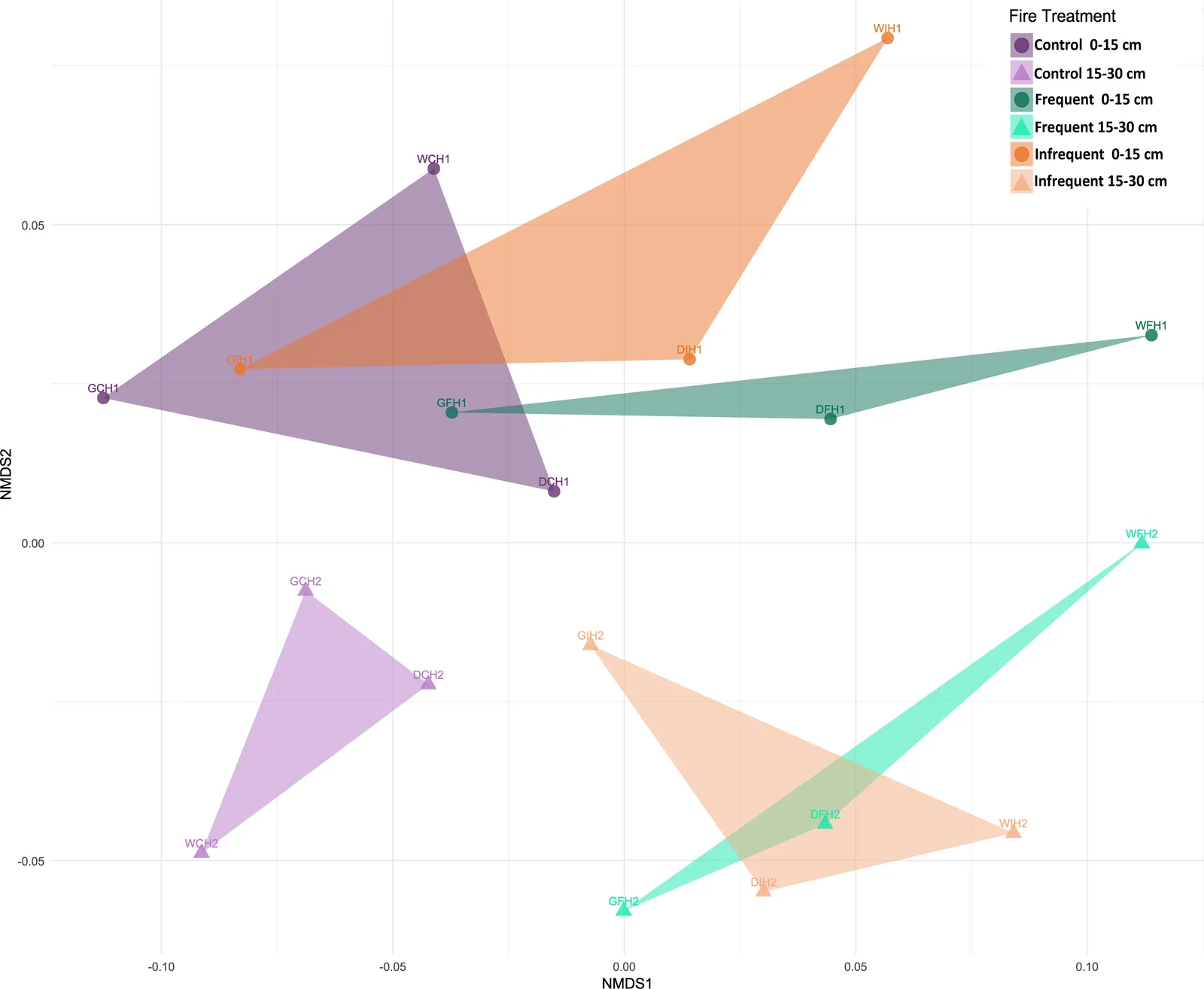
NMDS ordination based on Bray-Curtis dissimilarity of nitrogen-cycling gene composition across all samples. Soil depths of 0–15 and 15–30 cm are represented by circles and triangles, respectively. Control treatments are indicated in purple, infrequent in orange, and frequent in green. Sample names are labeled, with descriptions in Table S3. *R*^2^ = 0.994 and stress = 0.08.

Given the incomplete recovery of community members through metagenomic binning, we employed read-based mapping of N-cycling genes to capture changes in the functional potential across the broader, largely unassembled fraction of the metagenome (Table S24). Unlike the community analysis, variation in N-cycling gene composition was significantly explained by both fire treatment and depth (PERMANOVA, Table S25: *F =* 2.8*, R*^2^ = 0.26*, P* = 0.008, and*, F* = 3.7*, R^2^* = 0.17*, P* = 0.005, respectively) (Fig. 3). This is consistent with prior work in post-wildfire soils showing that fire can significantly modulate N-cycling functional potential (103, 104, 109). More broadly, our findings suggest a decoupling of functional gene distributions from the overall community structure—a pattern increasingly recognized in metagenomic studies, in which the overall microbial community composition may appear stable even as functional potential, at the gene level, diverges significantly across treatments or between distinct sites (110–112). This effect has been attributed to environmental selection for traits that are broadly distributed across diverse taxa (110, 111)—consistent with our observation that certain MAGs shift in abundance, while higher-level taxonomic assignments remain stable—as well as to the substantial pangenomic diversity that can be harbored within a single genus or even species (113–115).

Additionally, there was a significant (*P.*adj < 0.05) and moderate-to-strong positive correlation (Spearman’s rho > 0.60) between NO_3_-N concentrations and the abundances of the dissimilatory nitrate reduction (NO_3_^−^ → NO_2_^−^) and denitrification (defined here as NO_2_^−^ → NO → N_2_O → N_2_) marker genes, specifically *napABCDEH* (encoding the periplasmic nitrate reductase and its accessory proteins, NO_3_^−^ → NO_2_^−^), *narGHIJ* (respiratory nitrate reductase and its accessory proteins, NO_3_^−^ → NO_2_^−^), *nirK* (copper-containing nitrite reductase, NO_2_^−^ → NO), *nirT* (electron transfer protein to the nitrite reductase), *norB* (nitric oxide reductase, NO → N_2_O), and *nosZ* (nitrous oxide reductase, N_2_O → N_2_) (Table S26). These results suggest that the depletion of bioavailable NO_3_-N in fire-managed soils may be a driver of these shifts in N-cycling genes.

**Fig 4:**
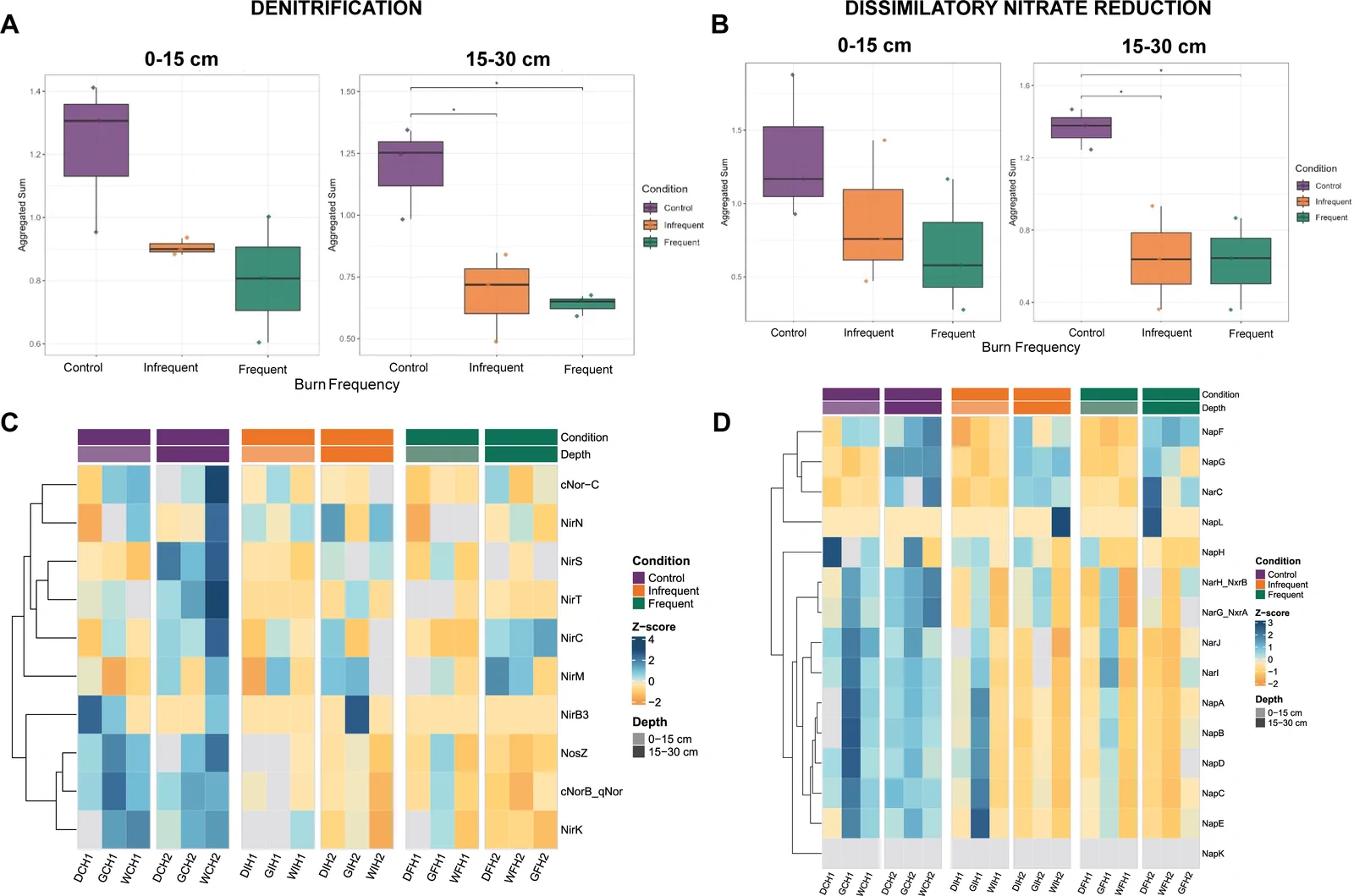
(**A and B**) Box-and-whisker plot of the sum of all genes (in EFPKG) in the denitrification (NO_3_^2−^→NO_2_^2−^→NO→ N_2_O→N_2_) (**A**), and dissimilatory nitrate reduction (NO_3_^2−^→NO_2_^2−^) (**B**) pathways from each stand at both soil depths. Gene data were summed by pathway. Significance was determined using a one-way ANOVA with a Tukey’s HSD *post hoc* test. Adjusted *P*-values were used to determine significance (*P.*adj < 0.05): *, *P.*adj < 0.05; **, P.adj < 0.01; ***, P.adj < 0.001; no stars, no significance. Details on the genes grouped in each pathway are shown in Table S4, while the pathways themselves are visualized in Fig. S2. (**C and D**) Normalized heatmap of genes present within the denitrification (**C**) and dissimilatory nitrate reduction pathways (**D**). Each row represents an individual gene, and each column represents a sample, grouped by burn frequency treatment (control, infrequent, and frequent) and soil depth (0–15 cm, lighter shading; 15–30 cm, darker shading). Gene abundance values were normalized by *Z*-score across samples, such that each value reflects the number of standard deviations above or below the mean abundance for that gene across all samples. Blue indicates above-average abundance (positive *Z*-score), yellow-orange indicates below-average abundance (negative *Z*-score), and gray cells indicate an abundance of zero. Completely grayed-out rows indicate genes for which no data were available across any sample.

We next examined the specific pathways within the N-cycle that were responsive to fire treatment (Fig. 4A and B; Fig. S19 to S25), and like the correlation analysis above, we observed decreases (one-way ANOVA with Tukey’s HSD*, P.*adj < 0.05) in the abundances of the denitrification (Fig. 4A) and dissimilatory nitrate reduction pathways (Fig. 4B) at the 15- to 30-cm depth in both fire treatments relative to controls. Together, these two pathways contribute to trace nitrogen gas fluxes, including nitrous acid (HONO) and nitric oxide (NO) (precursors to tropospheric ozone, a greenhouse gas) and the greenhouse gas nitrous oxide (N_2_O) (116, 117). Although trends in the same direction were observed at the 0- to 15-cm depth, these differences did not survive false discovery rate correction ( *P.*adj > 0.05). The lack of significant effects at the shallower depth may reflect the greater variability inherent in surface soils, which are more directly exposed to environmental heterogeneity, thereby reducing the statistical power to detect significant treatment effects. Within these pathways at the 15- to 30-cm depth, the specific genes that significantly decreased in abundance included *napA*, *nirK*, *norB*, and *nosZ* (Fig. 4C and D)—all of which were also identified as positively correlated with NO_3_-N concentrations. We also observed a significant increase in the anaerobic ammonium oxidation (NH_4_^+^ + NO → N_2_) pathway at the 0- to 15-cm depth (Fig. S19), although this finding is difficult to interpret: only a single, dedicated pathway gene (*hzsB*, encoding the hydrazine synthase beta subunit) was detected and only in about half of the samples at abundances orders of magnitude below those of almost all other nitrogen-cycling genes quantified here (Table S24). Finally, we observed no differences between treatments in the abundances of ammonium oxidation (NH_4_^+^ → NO_2_^−^, Fig. S20), nitrogen fixation (N_2_ → NH_4_^+^, Fig. S21), assimilatory nitrate reduction (NO_3_^−^ → NO_2_^−^, Fig. S22), ammonification (NO_2_^−^ → NH_4_^+^, Fig. S23), nitrite assimilation (NO_2_^−^ → NH_4_^+^, Fig. S24), and urea degradation (NH_2_CONH_2_ → NH_4_^+^, Fig. S25) pathways. As these pathways are all linked to ammonium flux (118), the stable ammonium concentrations across treatments (Fig. 2B) may explain the absence of detectable shifts.

Across multiple fire-affected ecosystems, studies have generally reported increases in the genetic potential for key nitrogen-cycling processes—particularly nitrification, denitrification, and nitrogen fixation—in the years following wildfire. In mixed-conifer forests of the Sierra Nevada (USA), Dove and colleagues found increased genetic potential for both denitrification and nitrification at the soil surface (0–5 cm) at 4 and 25 years post-fire, with a dip in both at 13 years, while nitrogen-fixing genetic capacity was elevated at all three time points (103). This pattern was echoed following a chaparral wildfire in Southern California, where Barriga and colleagues observed increases in the genetic potential for both nitrification and denitrification at the soil surface (0–10 cm) within the first year post-fire (119), and in an Alaskan boreal forest, where Taş and colleagues similarly reported increases in denitrification genes across a range of sample depths (0–40, 40–60, and 60–100 cm) 7 years post-fire (120). Not all results reported in the literature, however, align with this trend. In the rhizosphere of a Mediterranean forest in Spain, 3 years post-wildfire, burned rhizosphere samples showed increased abundances of nitrogen-fixation genes but decreased abundances of denitrification genes (121). This divergence may partly reflect the distinct microbial community selected for in the rhizosphere relative to that in bulk soil, which complicates direct comparisons with bulk-soil measurements reported by us and others. Taken together, the wildfire literature begins to point to a coherent mechanism: post-fire pulses in NO_3_-N drive increases in the genetic capacity for denitrification, likely by expanding substrate availability for denitrifying communities. The APBP, however, presents a contrasting picture. As described earlier, prescribed fire may generate more modest NO_3_-N pulses than wildfire (94), while simultaneously increasing the rate of NO_3_-N loss through erosion and leaching (97), thereby reducing substrate availability for denitrification.

### Taxonomic and genomic contexts of denitrification genes

Overall, the genes that were differentially abundant in our read-based analysis are largely missing from the recovered MAGs, suggesting that they are distributed across many low-abundance genomes that we failed to recover. Of the 19 MAGs harboring genes in these pathways, only one was differentially abundant—greater in frequently burned versus control stands: *Polyangiaceae* Bin.013.GFH2. *Polyangiaceae* Bin.013.GFH2 encodes *nirB* (NO_2_^−^ → NH_4_^+^), which did not differ in abundance across treatments in our earlier analyses. Among non-differentially abundant MAGs, *nirK* was detected in three nitrifier MAGs (*Nitrosopolaris rasttigaisensis* Bin.013.WCH2, *Nitrosopolaris* Bin.005.DIH2, and *Nitrospiraceae* Bin.019.DFH2); *norB* was also detected in Bin.019.DFH2, *nirK* and *nosZ* were found in *Gemmatimonadaceae* Bin.004.FCH1; and *nirK* was detected in *Ermiobacteraceae* Bin.002.GIH1. *napA* was absent from all MAGs.

We further examined the taxonomic assignment of the metagenomic reads mapping to the differentially abundant denitrification genes described above. At both depths, a large proportion of mapped reads for *napA*, *nirK*, *nosZ*, and *norB* were taxonomically assigned to the *Proteobacteria*, with contributions from *Bacteroidetes* (*norB* and *nosZ*), *Acidobacteria* (*qnor*), *Actinobacteria* (*nirK* and *norB*), *Chloroflexi* (*nirK*), *Nitrospirae* (*nirK*), *Chlamydiae* (*qnor*), and *Planctomycetes* (*norB*) (Fig. S26 and S27; Table S26). A small number of the *nirK* reads were attributed to the *Thaumarchaeota*. The differences in the taxonomic profiles of these reads suggest possible metabolic partitioning among denitrifiers at our site. This “division-of-labor" among denitrifiers has been reported in various other systems (122–124). Indeed, none of our MAGs encoding denitrification-related genes appear to be complete denitrifiers, although gaps in the assemblies complicate this interpretation. Compared with soils from the control stands, prescribed fire reduced the relative abundances of *Proteobacteria*-associated *napA* and *nirK* reads. In contrast, relative abundances of *Proteobacteria*-associated *nosZ* increased, while relative abundances of *nosZ* reads associated with *Bacteroidetes* declined. Trends were similar across both depths. Together, these shifts indicate that long-term fire management may also restructure the taxonomic partitioning of dissimilatory nitrate reduction/denitrification potential.

### Conclusions, limitations, and future directions

Despite extensive research on how wildfire affects soil microbiomes (101, 119, 125–135), the impacts of long-term prescribed fire regimes on microbial communities—and, particularly, their functional profiles—remain understudied. Prior work suggests that a single prescribed burn may have limited, short-lived effects on microbial taxonomic composition relative to more intense and severe wildfire—with detectable changes generally disappearing within weeks to months (12–14, 136). However, repeated prescribed burns have been associated with altered community composition in the interval between burns (17–25). In contrast, we observed no fire treatment-dependent changes in the overall community composition of both prokaryotes and fungi at the APBP, which we attribute to longer fire return intervals than in these prior studies (17, 20). Time since fire may also influence community development, although this is difficult to disentangle in earlier work, as time since fire and burn interval were identical in those studies. Notably, our burned sites did span a range of time since fire (1–5 years), and our ordinations showed no clustering by this metric—although our sample size limits the strength of this observation, as time since fire was not a factor in our study design.

We did identify several MAGs that were differentially abundant between the control and frequently burned sites, suggesting treatment-specific community differentiation at the genome scale. Furthermore, our functional gene analysis revealed that repeated prescribed fire management led to a persistent restructuring of microbial N-cycling potential. Most notably, we observed a sustained reduction in denitrification potential, which links repeated prescribed fire to long-term modulation of trace nitrogen gas fluxes relevant to climate change—HONO, NO, and N_2_O (116, 117). These functional profile shifts were accompanied by fire-dependent changes in soil nitrogen (specifically %TN and NO_3_-N), consistent with prior reports linking denitrifier abundance to soil nitrogen availability (137, 138). While fire is generally associated with increased emissions of these trace nitrogen gases (139), our findings suggest that repeated prescribed burning might have the opposite effect in some systems. This is consistent with a small body of evidence emerging in the literature: in a Sierra Nevada chaparral shrubland, prescribed burns at 4-year intervals reduced N_2_O fluxes relative to longer intervals (20 years) or fire suppression (140). Similarly, NO emissions decreased under biennial prescribed burns compared with fire-suppressed plots in a South African savanna (141). Thus, prescribed fire regimes may dampen microbial nitrogen cycling, with potential consequences for ecosystem nitrogen retention and climate change. However, activity measurements are required to test this hypothesis.

Overall, our study highlights the need for functional analyses of microbial communities in systems managed with prescribed fire, as fire-driven changes may be more subtle and primarily at the genome or gene level rather than in broad taxonomic composition. This would enable comparisons to research assessing community responses following more intense and severe wildfires, a topic of significant interest as prescribed fires aim to reduce the frequency and severity of wildfires. However, whether our findings generalize across different ecosystems managed with prescribed burns remains an open question for further exploration. Finally, a key limitation is our single time point for analysis. Although time since fire varied across our stands by an ecologically appropriate 1–5 years (6, 142), our study was designed to more generally assess the effects of prescribed fire frequency and did not involve intentional replication at various times since fire. Future work should use repeated sampling throughout the burn interval and incorporate direct measurements of trace nitrogen gas emissions to better constrain how prescribed fire regimes modulate microbial nitrogen cycling over time.

## Data Availability

All geochemical data and site metadata are available in the Supplemental information files. All sequencing data used here are associated with BioProject PRJNA1269515. Raw sequencing data are available through NCBI Sequence Read Archive (SRA) via accession numbers SRX28992350–SRX28992355; SRX29211976–SRX29211981; and SRX29333943–SRX29333948.

## SUPPLEMENTAL MATERIAL

Supplemental Information msystems.00852-26-s0001.pdf Supplemental methods; Tables S2, S4, S6, S25, and 26; Figures S1–S27.

Supplemental Tables msystems.00852-26-s0002.xlsx Tables S1, S3–S5, S7–S25, and S27.

ASM does not own the copyrights to Supplemental Material that may be linked to, or accessed through, an article. The authors have granted ASM a non-exclusive, world-wide license to publish the Supplemental Material files. Please contact the corresponding author directly for reuse.
