# Supplementary material for: Prescribed burns drive lasting changes in soil nitrogen cycling and microbial function
Source: mSystems. 2026 Sep 21;11(10):e00852-26. doi: 10.1128/msystems.00852-26 (PMC13712880; doi:10.1128/msystems.00852-26)
Supplement: Supplementary file 1 [file msystems.00852-26-s0001.pdf]

Supporting Information For:

Prescribed Burns Drives Lasting Changes in Soil Nitrogen Cycling and Microbial Function

---

Supplemental Methods

Supplementary Figures and Tables

Table S1. Excel sheet

Table S2. This document

Table S3. Excel sheet

Table S4. This document

Table S5. Excel sheet

Table S6. This document

Tables S7-S25. Excel sheet

Tables S25-S26. This document

Table S27. Excel sheet

Figures S1-S27. This document

References for Supplemental Information

Supplemental Materials

APBP Site Description: The APBP (42.71 °N, 73.86 °W) landscape is dominated by a relatively open canopy of pitch pine (*Pinus rigida*) and is interspersed with dense thickets of scrub oak (*Quercus ilicifolia* and *Q. prinoides*). It also contains a diverse understory marked by shrubs such as blueberry (*Vaccinium spp.),* black huckleberry (*Gaylussacia baccata),* and New Jersey tea (*Ceanothus americanus*), as well as herbaceous species like big bluestem (*Andropogan gerardii*), little bluestem (*Schizachyrium scoparium),* and wild lupine (*Lupinus perennis*) (1–5). This plant community is predicated upon the existence of the sandy, highly permeable, soils that exist at the site—primarily Colonie Loamy Fine Sand and Elnora soils (1, 3, 6). These acidic soils are of glaciofluvial origin and create a low-nutrient dry surface – conditions that promote fire – with a more saturated and better nourished sublayer (1, 3, 7, 8). More details on the soil can be found in **Fig. S1**.

Functional Gene Analysis: Translated reads were mapped to a curated database of nitrogen-cycle enzymes (~22,000 proteins across 57 protein families from SEED (9) and KEGG (10)) using DIAMOND (11), generating a taxonomically resolved functional profile. Database hits were filtered based on alignment length and amino acid identity (AAI), using protein family-specific thresholds. Mapped read counts, taxonomic, and functional assignments were collated for all hits. To enable quantitative comparisons across genes and samples, raw counts of the mapped fragments (N_f) were normalized by effective gene length (EGL), sequencing depth, and estimated genome equivalents (GE). Normalized abundances are expressed as EFPKG (effective fragments per kilobase per genome equivalent):

$$EFPKG = (N_f) / (EGL) / (GE)$$

The effective gene length (EGL) is calculated as:

$$EGL = GL + F - 2 \times AL + 1$$

Where GL is the gene length, AL is the minimum alignment length, and F is the average fragment length. GEs are used to normalize for differences in community genome size and sequencing depth, calculated as:

$$GE = \text{total reads} / \text{average genome size}$$

The average genome size for each sequencing library is estimated using MicrobeCensus (12). Pathway abundances are determined by summing the EFPKG values for multiple genes in a particular pathway.

SUPPLEMENTAL FIGURES AND TABLES

Table S2. Stand Fire History

Stand	Sample ID Prefix	Fire Frequency	Total Number of Fires Since 1991	Average Fire Interval (1991-2024)	Fire Dates*
Draperies	DF	Frequent	4	8.25	1994, 2005, 2018, 2023
Witches Hat	WF	Frequent	4	8.25	1999, 2005, 2017, 2021
Genus	GF	Frequent	5	6.6	1995,1996, 2004, 2019, 2023,
Dandy	DI	Infrequent	2	16.5	2006, 2019
West Overlook	WI	Infrequent	3	16.5	1999 (escaped prescribed burn that turned into a wildfire), 2017, 2022
Gandalf	GI	Infrequent	2	16.5	1997, 2022
Diversity	DC	Control	0	*n/a*	*n/a*
Hydro	WC	Control	0	*n/a*	*n/a*
Gulch	GC	Control	0	*n/a*	*n/a*

*Unless otherwise marked as a wildfire, all fires were prescribed burns conducted by the APBP

Gene	Description of enzyme	Pathway	Source
	**Ammonification**	**$NO_2^- \rightarrow NH_4^+$**	
nrfA	nitrite reductase	Ammonification	(13)
nrfC *	protein NrfC; predicted to contain 4 [4Fe-4S] clusters	Ammonification	(14, 15)
nrfD *	protein NrfD; membrane-bound quinol oxidase	Ammonification	(14, 15)
nrfH *	tetraheme c-type cytochrome nitrite reductase	Ammonification	(16–18)
	**Ammonium Oxidation**	**$NH_4^+ \rightarrow NO_2^-$**	
amoA_pmoA	methane/ammonia monooxygenase subunit A; inferred enzyme active site	Ammonium Oxidation	(19)
amoB_pmoB	methane/ammonia monooxygenase subunit B	Ammonium Oxidation	(19)
amoC_pmoC	methane/ammonia monooxygenase subunit C	Ammonium Oxidation	(19)
hao	hydroxylamine dehydrogenase; nitrite forming	Anaerobic Ammonium Oxidation	(20, 21)
	**Anaerobic Ammonium Oxidation**	**$NO + NH_4^+ \rightarrow N_2H_4 \rightarrow N_2$**	
hzo	hydrazine dehydrogenase	Anaerobic Ammonium Oxidation	(21)
hzsB	hydrazine synthase subunit B	Anaerobic Ammonium Oxidation	(21, 22)
	**Denitrification**	**$NO_2^- \rightarrow NO \rightarrow N_2O \rightarrow N_2$**	
cnorB_qnor	nitric-oxide reductase; c-type cytochrome/quinol dependent	Denitrification	(23)
cnor-C	nitric-oxide reductase subunit; heterodimer c-type cytochrome	Denitrification	(24)
nirB3 *	cytochrome c-552 precursor NirB; large subunit	Denitrification	(25)
nirC *	cytochrome c55X	Denitrification	(26)
nirK	copper containing nitrite reductase; NO-forming	Denitrification	(27, 28)
nirM *	periplasmic cytochrome c551	Denitrification	(29)
nirN *	nitrite reductase associated c-type cytochrome; dihydro-heme d1 dehydrogenase	Denitrification	(20, 30)
nirS	cytochrome cd1 nitrite reductase; hydroxylamine reductase; NO-forming	Denitrification	(27, 28)
nirT *	tetraheme cytochrome c-type protein	Denitrification	(18, 31)
nosZ	nitrous-oxide reductase; catalytic subunit	Denitrification	(23)
	**Nitrate Assimilatory Reduction**	**$NO_3^- \rightarrow NO_2^-$**	
nasA	assimilatory nitrate reductase large subunit	Nitrate Assimilatory Reduction	(32)
nasB	assimilatory nitrate reductase NADH oxidase subunit	Nitrate Assimilatory Reduction	(32)
	**Nitrate Dissimilatory Reduction**	**$NO_3^- \rightarrow NO_2^-$**	
napA	molybdoprotein periplasmic nitrate reductase precursor	Nitrate Dissimilatory Reduction	(33, 34)
napB	di-heme periplasmic nitrate reductase cytochrome c550-type subunit	Nitrate Dissimilatory Reduction	(33)
napC	tetraheme cytochrome c-type protein	Nitrate Dissimilatory Reduction	(18, 31)
napD *	periplasmic nitrate reductase component	Nitrate Dissimilatory Reduction	(18)
napE *	periplasmic nitrate reductase component	Nitrate Dissimilatory Reduction	(35)
napF *	periplasmic nitrate reductase; ferredoxin-type protein NapF	Nitrate Dissimilatory Reduction	(18)
napG *	periplasmic nitrate reductase; ferredoxin-type protein NapG	Nitrate Dissimilatory Reduction	(18)
napH *	periplasmic nitrate reductase; polyferredoxin NapH	Nitrate Dissimilatory Reduction	(36)
napL *	periplasmic nitrate reductase component NapL; ε-proteobacteria	Nitrate Dissimilatory Reduction	(35)
narC *	respiratory nitrate reductase subunit; cytochrome b-561	Nitrate Dissimilatory Reduction	(37)
narG_nxrA	nitrate reductase/nitrite oxidoreductase; alpha subunit	Nitrate Dissimilatory Reduction	(23)
narH_nxrB	nitrate reductase/nitrite oxidoreductase; beta subunit	Nitrate Dissimilatory Reduction	(23)
narI	respiratory nitrate reductase gamma chain	Nitrate Dissimilatory Reduction	(23)
narJ *	nitrate reductase molybdenum cofactor assembly chaperone of NarJ/NarW	Nitrate Dissimilatory Reduction	(23)
	**Nitrite Assimilation**	**$NO_2^- \rightarrow NH_4^+$**	
nasI *	assimilatory nitrate reductase; electron transfer subunit; Clostridium genus	Nitrite Assimilation	(37)
nasJ *	assimilatory nitrate reductase; NADH oxidase subunit; Clostridium genus	Nitrite Assimilation	(38)
nirA	ferredoxin-nitrite reductase	Nitrite Assimilation	(39)
nirB	nitrite reductase; NADH; large subunit	Nitrite Assimilation	(40, 41)
nirD	nitrite reductase; NADH; small subunit	Nitrite Assimilation	(40, 41)
nirU *	copper containing assimilatory nitrite reductase; putative NADH oxidase subunit	Nitrite Assimilation	(42)
	**Nitrogen Fixation**	**$N_2 \rightarrow NH_4^+$**	
anfG_vnfG *	nitrogenase delta subunit	Nitrogen Fixation	(43)
nifB	nitrogen fixation protein NifB	Nitrogen Fixation	(44)
nifD_anfD_vnfD	nitrogenase molybdenum-iron protein alpha chain; NifD/AnfD/VnfK	Nitrogen Fixation	(45, 46)
nifH_anfH_vnfH	nitrogenase reductase; maturation iron protein NifH/AnfH/VnfH	Nitrogen Fixation	(45–47)
nifK_anfK_vnfK	nitrogenase molybdenum-iron protein beta chain; NifK/AnfK/VnfK	Nitrogen Fixation	(45, 46)
	**Urease**	**Organic N $\rightarrow NH_4^+$**	
ureA	urease subunit gamma	Urease	(48)
ureB	urease subunit beta	Urease	(48)
ureC	urease subunit alpha; houses catalytic nickel binding site	Urease	(48, 49)

* = not displayed in the associated pathway map

Table S4: Nitrogen cycling genes present in this study with corresponding functional annotations, organized by pathways as, defined by the Fama pipeline (https://iseq.lbl.gov/fama/reference/nitrogen/nitrogen/). A corresponding pathway map is shown in **Fig. S2**.

	Table S6. Averaged Soil Geochemistry Per Treatment + Depth (AVG ±SD)															
**Treatment**	**pH**	**% LOI**	**% TN**	**% TC**	**NO3-N***	**NH4-N***	**Ca***	**K***	**Mg***	**P***	**Al***	**Fe***	**Mn***	**Na***	**Zn***	**Acidity^**	**ECEC^**
**0-15 cm**																	
Frequent	5.3±0.2	3.2±0.8	0.09±0.03	1.6±0.5	1.1±0.8	7.4±5.2	234.7±160.1	50.3±28.1	20.7±7.4	1.4±0.6	84.7±18.0	3.8±0.5	6.3±3.8	4.6±4.7	1.1±0.1	1.1±0.2	2.6±0.7
Infrequent	5.3±0.2	4.1±1.4	0.10±0.02	1.7±0.3	1.3±1.4	9.8±5.0	147.7±90.6	40.7±23.2	14.3±5.6	2.2±0.8	103.7±40.1	4.5±1.3	5.6±1.9	7.8±0.6	1.7±0.4	1.3±0.5	2.3±0.3
Control	4.7±0.2	4.7±1.2	0.14±0.02	2.6±0.8	9.6±2.6	8.1±4.3	342.0±183.9	56.3±17.8	34.7±15.5	3.0±0.6	101.7±23.2	7.7±2.6	15.0±7.8	12.0±10.0	2.7±1.3	1.5±0.1	3.7±1.1
**15-30 cm**																	
Frequent	5.4±0.2	1.7±0.8	0.04±0.01	0.6±0.2	0.5±0.3	2.1±0.5	50.7±33.6	15.7±0.6	4.3±1.0	1.4±1.0	51.3±7.8	2.4±0.4	1.4±0.9	12.5±17.7	0.6±0.9	0.7±0.0	1.1±0.1
Infrequent	5.1±0.1	1.6±0.3	0.04±0.02	0.7±0.2	0.6±0.4	3.0±1.7	45.3±23.4	26.0±18.4	5.3±1.9	1.1±0.2	63.3±12.7	3.3±0.7	2.4±0.7	5.5±2.5	0.7±0.7	0.8±0.1	1.2±0.3
Control	5.1±0.1	2.0±0.6	0.05±0.01	0.9±0.4	2.3±0.3	2.9±2.1	90.3±30.0	36.3±18.5	11.7±3.2	2.1±0.9	69.3±1.5	3.4±1.0	5.0±3.1	11.5±5.9	1.0±0.6	0.9±0.0	1.6±0.2

*mg/kg
^ meq/100 g
Unprocessed data are available in **Table S3**.

Variable	***Df***	***Sum Of Sqs***	***R2***	***F***	***Pr (>F)***
Treatment	2	0.01555	0.25517	2.794904	**0.008**
Depth	1	0.010339	0.16966	3.71661	**0.005**
Treatment x Depth	2	0.001669	0.027379	0.299889	0.959
Residual	12	0.033383	0.54779		
Total	17	0.06094	1		

Table S25. PERMANOVA (Bray-Curtis dissimilarity) results showing the effects of Treatment (Frequent, Infrequent, Control), Depth (0-15cm, 15-30cm), and their interaction on the nitrogen-cycling gene composition. Significance was determined at $p < 0.05$.

Table S26. Correlations Between NO3⁻-N and Gene Abundances							
Gene Product	rho	p_value	p_adj	Gene Product	rho	p_value	p_adj
**NapA**	**0.9023**	0	0	NrfH	0.3659	0.1354	0.2401
**NosZ**	**0.8806**	0	0	NapL	-0.3643	0.1372	0.2401
**cNorB_qNor**	**0.8662**	0	0	NirB	0.3307	0.18	0.3012
**NapB**	**0.862**	0	0	UreA	-0.3276	0.1844	0.3012
**NapC**	**0.9044**	0	0	NifB	0.3163	0.201	0.3177
**NapD**	**0.8879**	0	0	NapG	-0.2429	0.3315	0.5076
**NapE**	**0.8693**	0	0	NasA	0.2346	0.3487	0.5178
**NarI**	**0.8506**	0	0	UreC	-0.216	0.3893	0.561
**NarJ**	**0.9106**	0	0	AmoA_PmoA	-0.2098	0.4034	0.562
**NarG_NxrA**	**0.7535**	0.0003	0.0015	UreB	-0.2057	0.4129	0.562
**NirK**	**0.739**	0.0005	0.0022	NirN	-0.184	0.4649	0.6157
**NarH_NxrB**	**0.6698**	0.0024	0.0098	NasJ	0.1145	0.6508	0.7986
**NirT**	**0.6327**	0.0048	0.0181	NirM	-0.1132	0.6546	0.7986
**NapH**	**0.6159**	0.0065	0.0216	NarC	-0.1116	0.6592	0.7986
HzsB	-0.6149	0.0066	0.0216	HAO	0.1085	0.6682	0.7986
**AnfG_VnfG**	**0.5913**	0.0097	0.0297	NirC	-0.0909	0.7199	0.8376
NrfC	-0.5354	0.022	0.0634	AmoB_PmoB	-0.0858	0.735	0.8376
NrfA	0.4775	0.0451	0.1228	NrfD	0.0735	0.7718	0.8443
NirU	0.4713	0.0483	0.1246	NirA	0.0724	0.7754	0.8443
NifD_AnfD_VnfD	0.4651	0.0518	0.1269	AmoC_PmoC	0.032	0.8996	0.9479
cNor-C	0.4589	0.0554	0.1293	NirS	0.0289	0.9092	0.9479
NifK_AnfK_VnfK	0.4403	0.0674	0.1501	NapF	-0.0217	0.9319	0.9513
NifH_AnfH_VnfH	0.4062	0.0944	0.2011	NasI	0.0058	0.9818	0.9818
NirB3	0.3924	0.1073	0.2191				
NasB	0.369	0.1318	0.2401				
NirD	0.3669	0.1342	0.2401				

*Significantly (p_adj < 0.05) positively correlated results are bolded

A

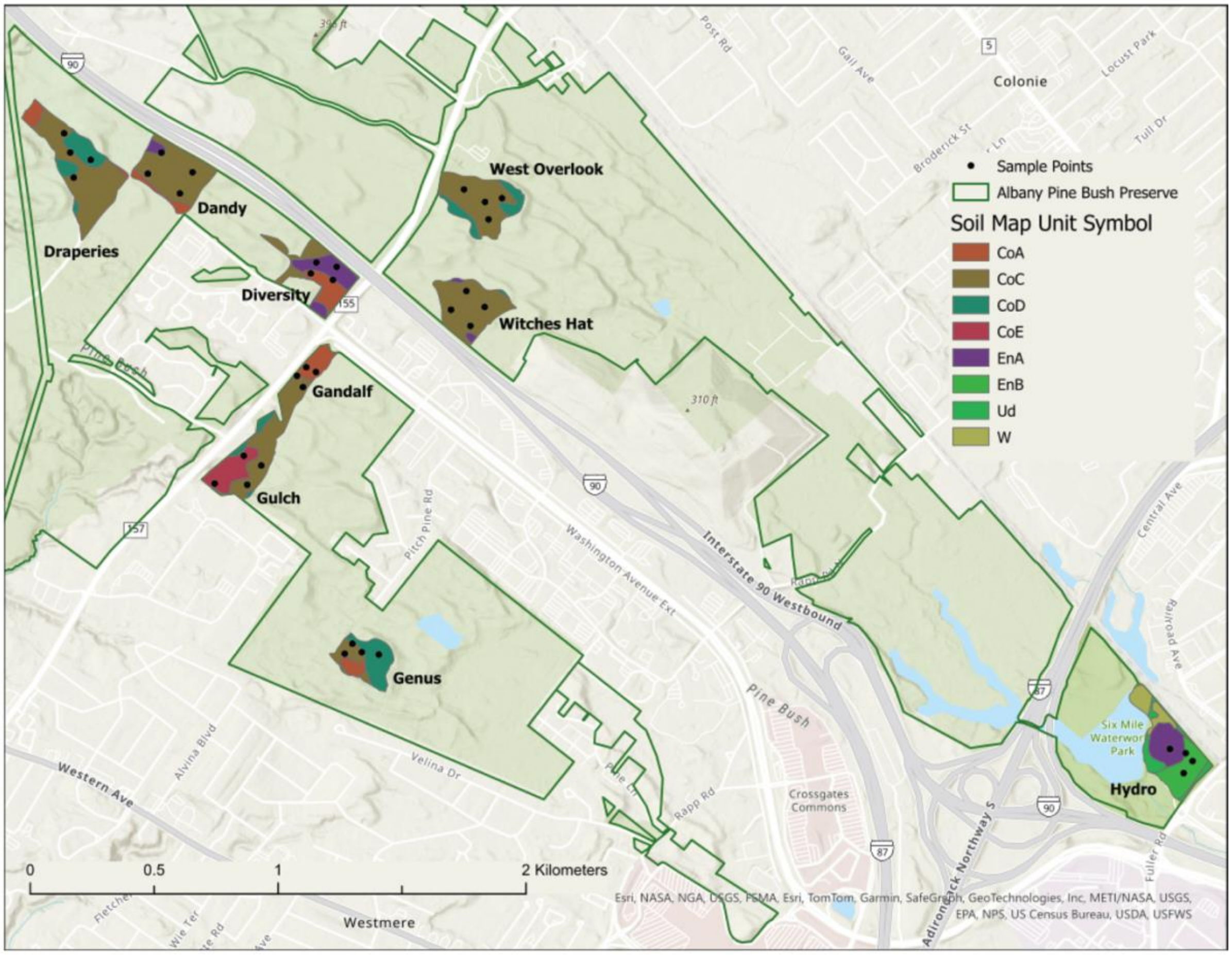

B

Soil Name	Symbol	Ecological Class(es)	Texture	Depth (in) (*cm*)	Sand (%)	Silt (%)	Clay (%)
Colonie loamy fine sand, 0 to 3 percent slopes	CoA	NRCS Forestland Site-Dry Outwash	loamy fine sand	0-7 (*0 - 17.78*)	71-89	1-29	1-2
				7-68 (*17.78 - 172.72*)	71-100	1-29	1-14
Colonie loamy fine sand, rolling	CoC	NRCS Forestland Site-Dry Outwash	loamy fine sand	0-7 (*0 - 17.78*)	71-89	1-29	1-2
				7-68 (*17.78 - 172.72*)	71-100	1-29	1-14
Colonie loamy fine sand, hilly	CoD	NRCS Forestland Site-Dry Outwash	loamy fine sand	0-7 (*0 - 17.78*)	71-89	1-29	1-2
				7-68 (*17.78 - 172.72*)	71-100	1-29	1-14
Colonie loamy fine sand, very hilly	CoE	NRCS Forestland Site-Dry Outwash	loamy fine sand	0-7 (*0 - 17.78*)	71-89	1-29	1-2
				7-68 (*17.78 - 172.72*)	71-100	1-29	1-14
Elnora loamy fine sand, 0 to 3 percent slopes	EnA	NRCS Forestland Site-Moist Outwash	loamy fine sand	0-11 (*0 – 27.94*)	71-89	1-29	2-10
			fine sand	11-27 (*27.94 - 68.58*)	71-100	0-29	1-14
Elnora loamy fine sand, 3 to 8 percent slopes	EnB	NRCS Forestland Site-Moist Sandy Outwash	loamy fine sand	0-11 (*0 – 27.94*)	71-89	1-29	2-10
			fine sand	11-27 (*27.94 - 68.58*)	71-100	0-29	1-14
Udipsamments, smoothed	Ud	*N/A*	coarse sand	0-70 (*0 - 177.8*)	86-100	1-14	0-2
Water	W	*N/A*	*N/A*	*N/A*	*N/A*	*N/A*	*N/A*

Figure S1: A. Map of the Albany Pine Bush Preserve (outline dark green) displaying soil types and corresponding locations within chosen plot. Sampling sites are denoted in black circles as determined by latitude and longitude coordinates. **B.** Soil taxonomy and descriptions for chosen APBP sites along with corresponding map unit symbol. NCRS denotes National Resource Conservation Service.

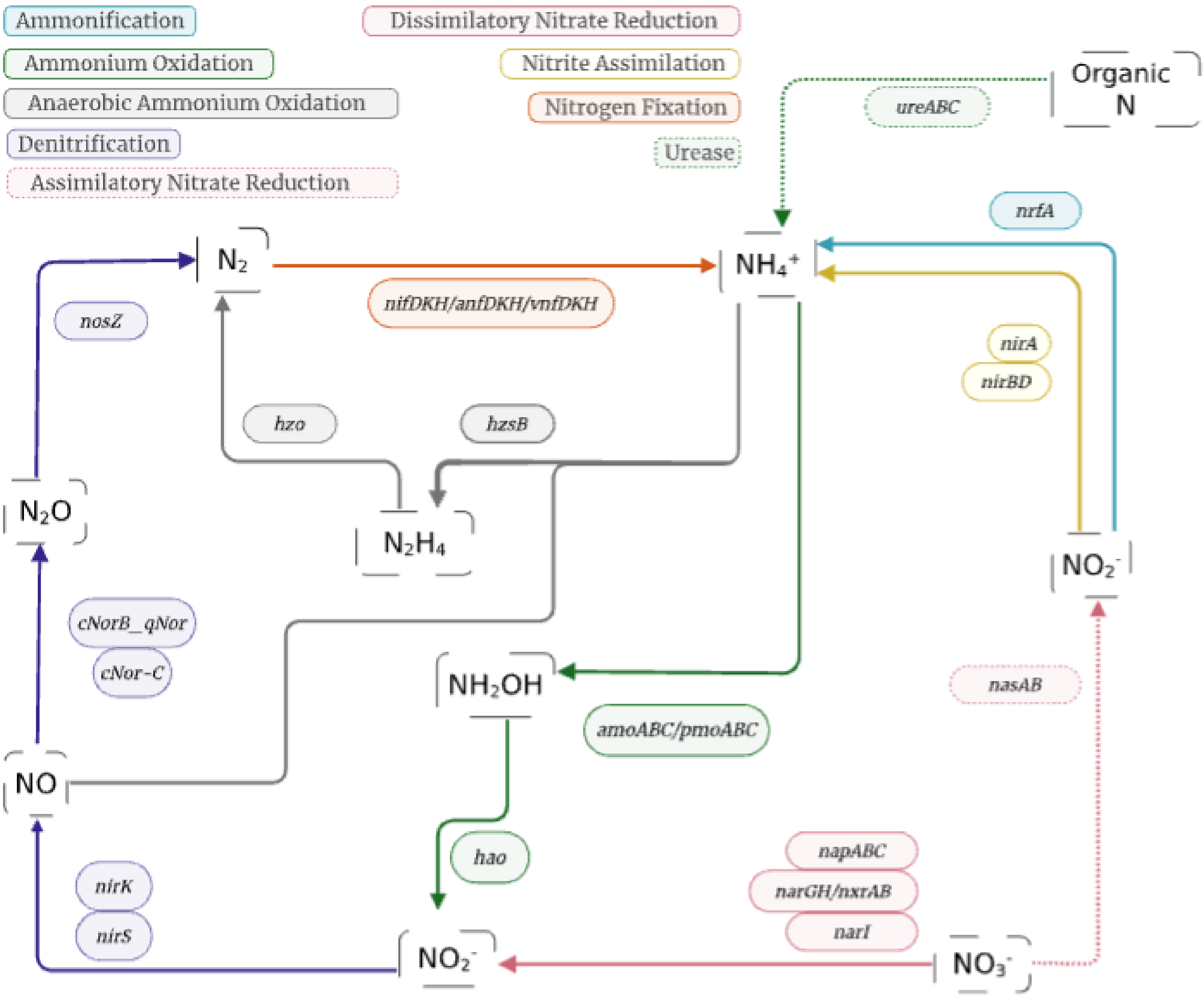

Figure S2: Nitrogen cycle (non-exhaustive) pathways with corresponding gene annotation as defined by Fama. All genes are italicized within oval boxes, pathway name, related gene, and arrows are all shown in the same color with arrows representing direction of pathway. NH_4^+: ammonium ion; N_2: dinitrogen gas; N_2O: nitrous oxide; NO: nitric oxide; NO_2^-: nitrite ion; NO_3^-: nitrate ion; NH_2OH: hydroxylamine; N_2H_4: hydrazine. For further information and genes encoding accessory proteins see **Table S4.**

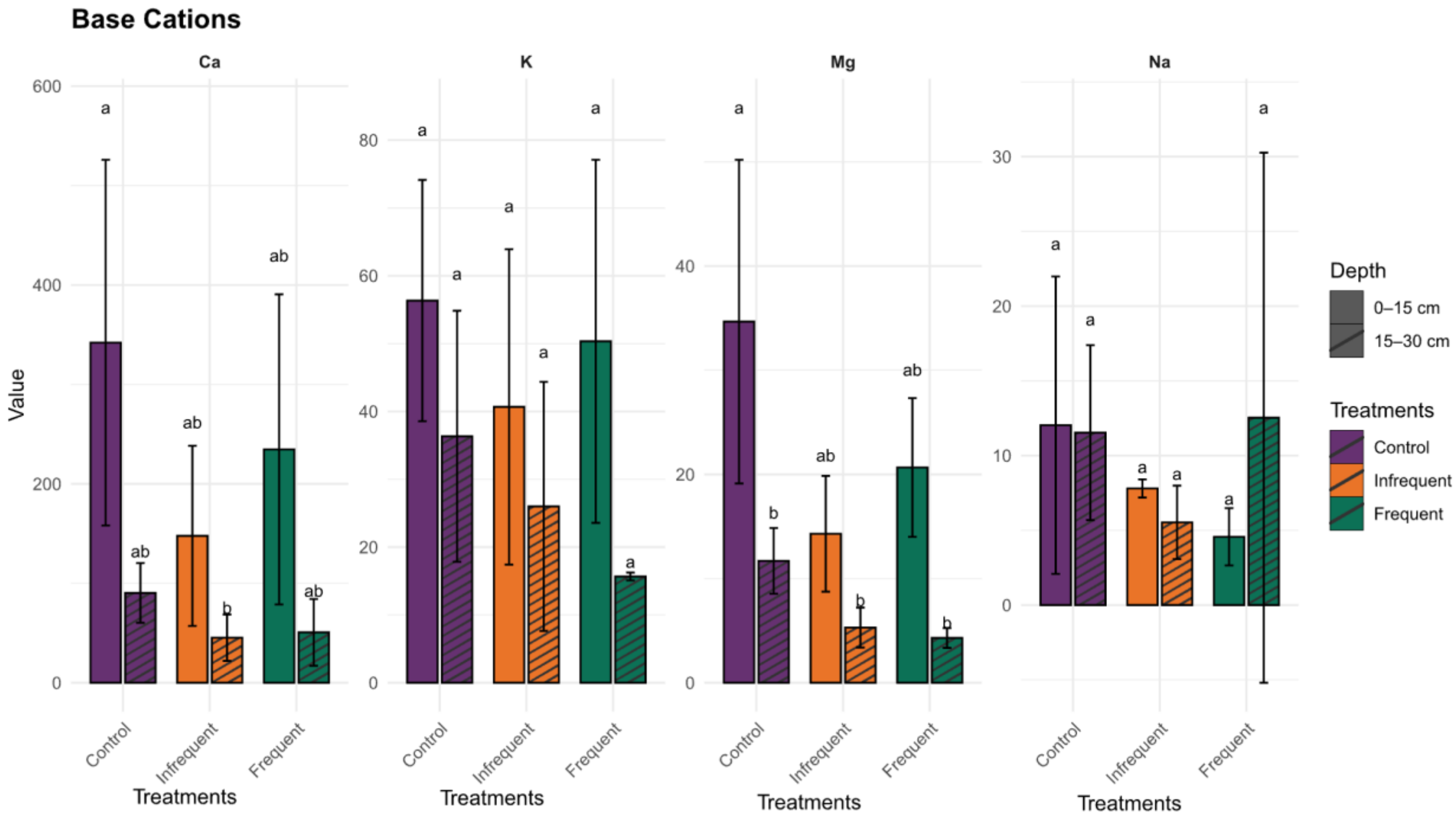

Figure S3: Averaged concentrations (±SD) of base cations of calcium (Ca, *mg/kg*), potassium (K, *mg/kg*), magnesium (Mg, *mg/kg*), and sodium (Na, *mg/kg*) across all fire treatments and soil depths. Solid and striped columns represent 0-15 and 15-30 cm in depth, respectively. Two-way Analysis of Variance (ANOVA) with post hoc Tukey Honestly Significant Difference performed. Letters above bars indicant significant differences amongst treatments within their respective depth ($p < 0.05$); bars sharing the same letter are not significantly different. Errors bars represent standard deviation (±SD).

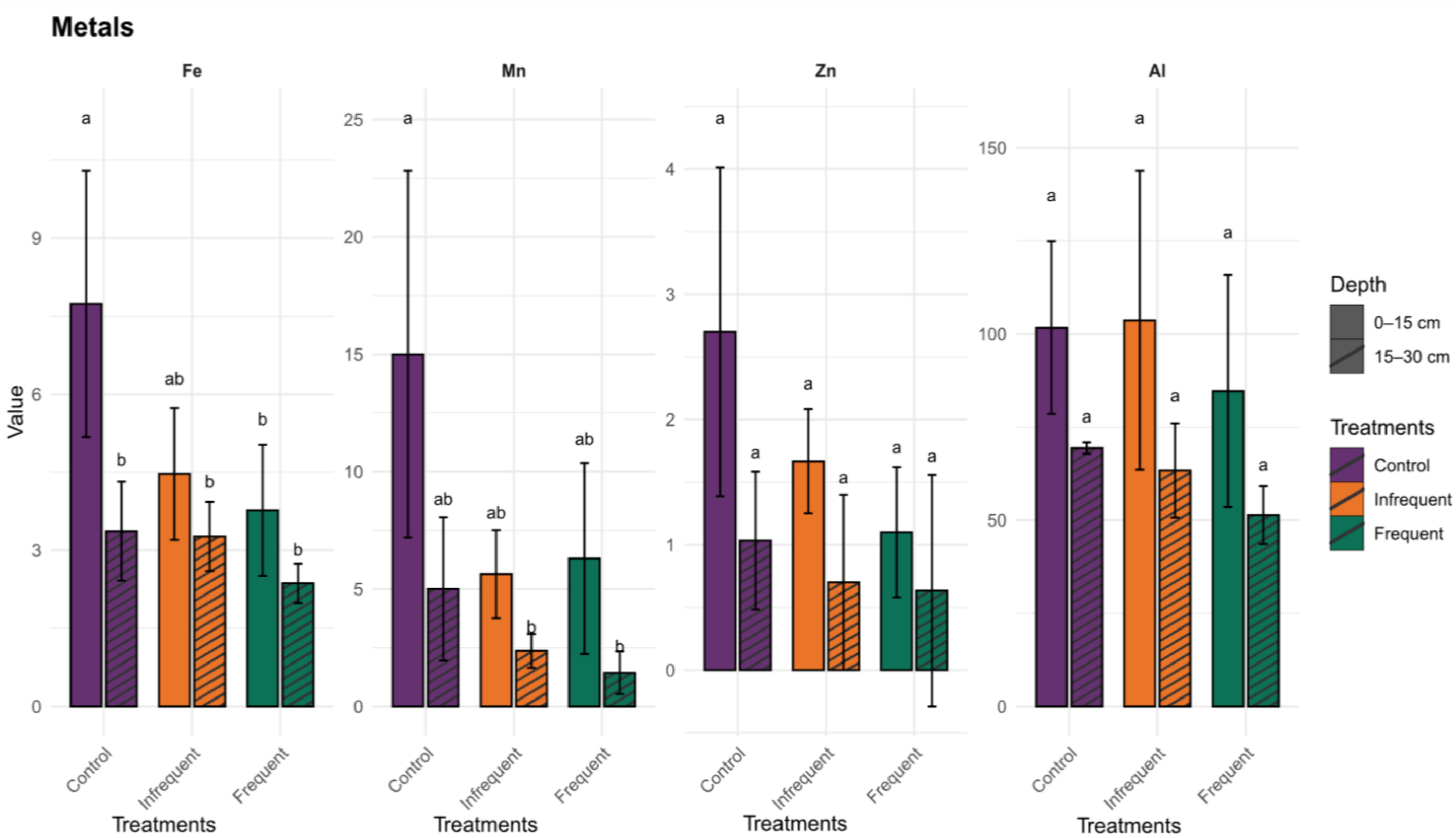

Figure S4: Averaged concentrations (±SD) of iron (Fe, *mg/kg*), manganese (Mn, *mg/kg*), zinc (Zn, *mg/kg*), and aluminum (Al, *mg/kg*) across all fire treatments and soil depths. Solid and striped columns represent 0-15 and 15-30 cm in depth, respectively. Two-way Analysis of Variance (ANOVA) with post hoc Tukey Honestly Significant Difference performed. Letters above bars indicant significant differences amongst treatments within their respective depth ($p < 0.05$); bars sharing the same letter are not significantly different. Errors bars represent standard deviation (±SD).

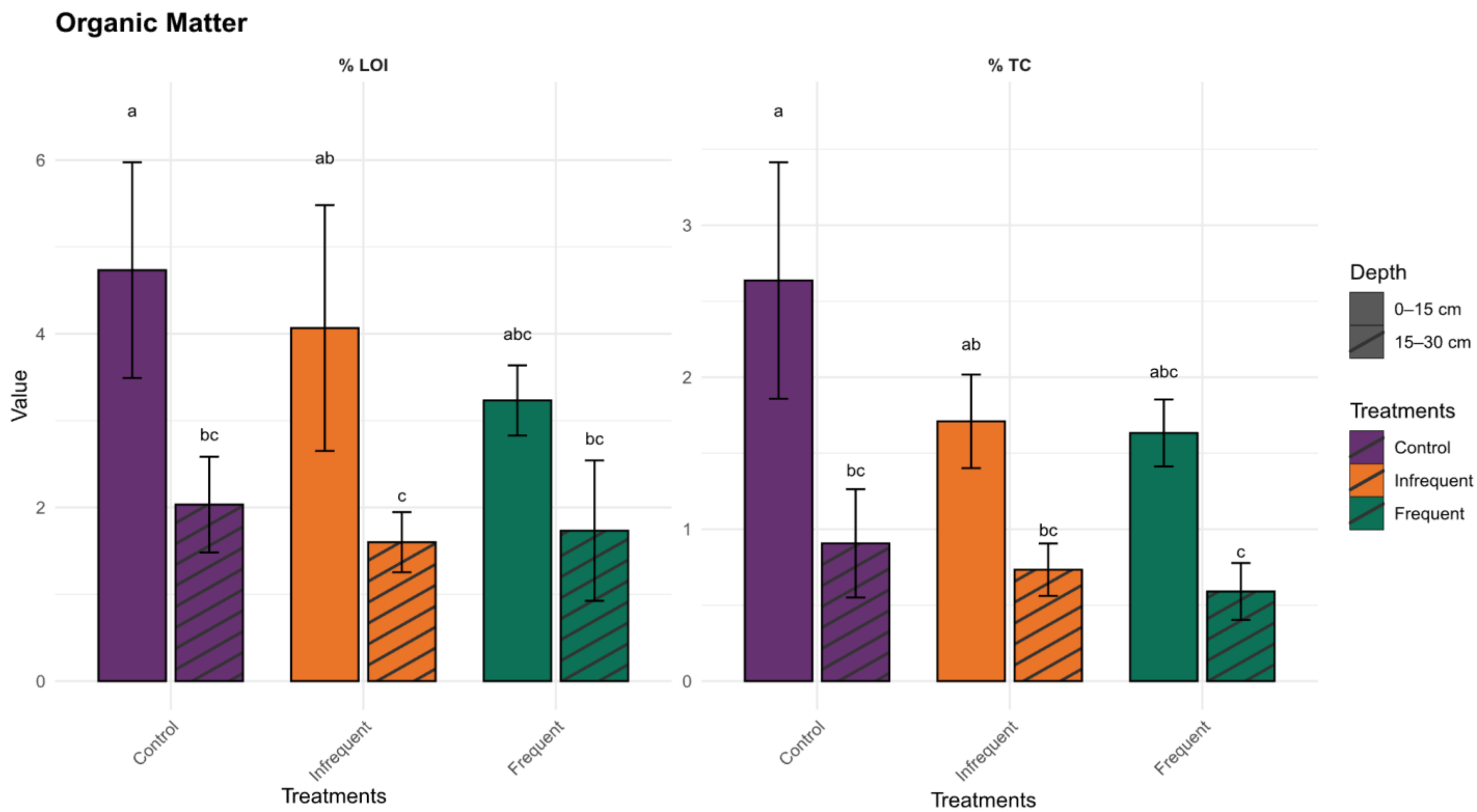

Figure S5: Averaged concentrations (±SD) of percent of organic matter lost on ignition (%LOI) and percent total carbon (%TC) across all fire treatments and soil depths. Solid and striped columns represent 0-15 and 15-30 cm in depth, respectively. Two-way Analysis of Variance (ANOVA) with post hoc Tukey Honestly Significant Difference performed. Letters above bars indicant significant differences amongst treatments within their respective depth ($p < 0.05$); bars sharing the same letter are not significantly different. Errors bars represent standard deviation (±SD).

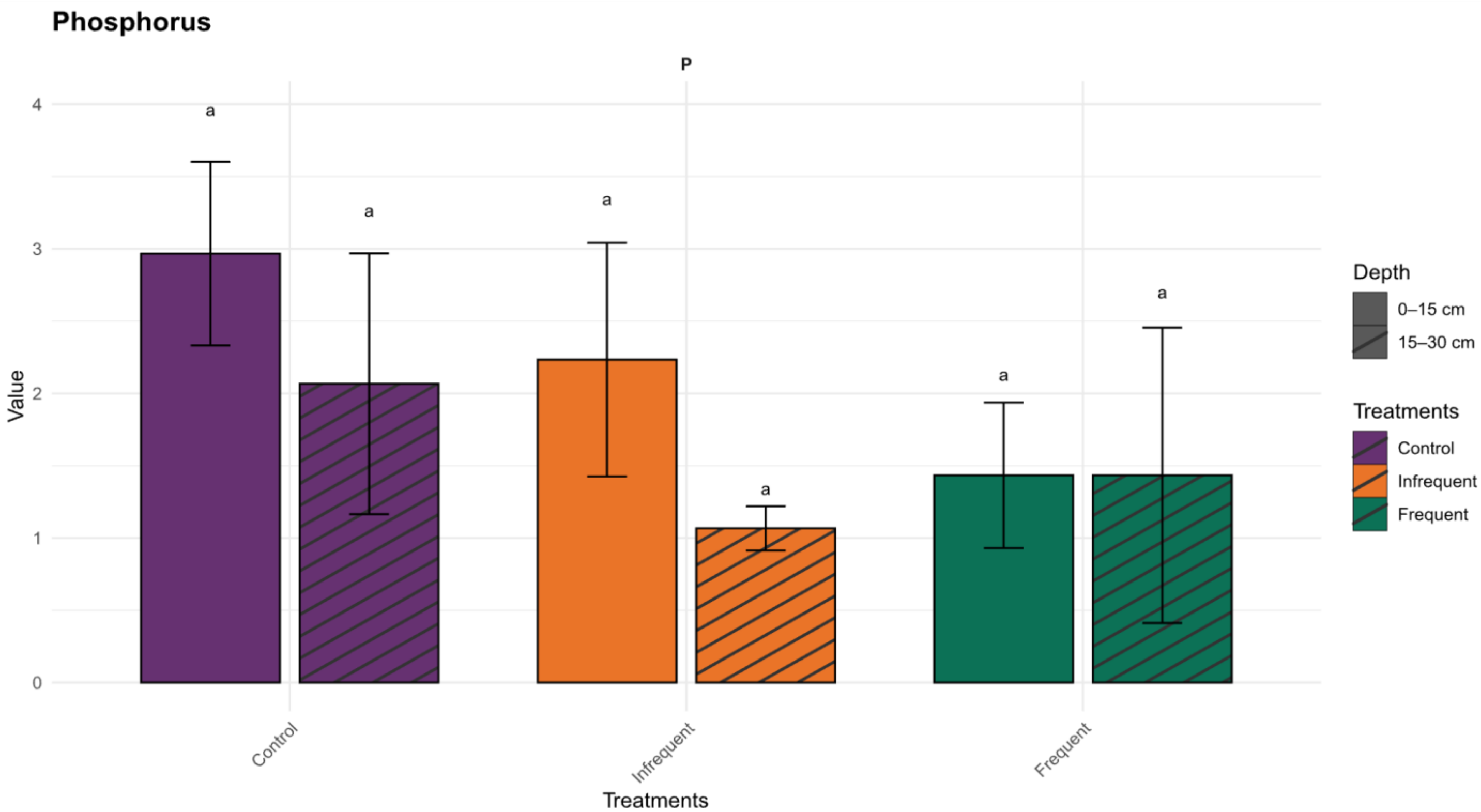

Figure S6: Averaged concentrations (±SD) of phosphorus (P, *mg/kg*) across all fire treatments and soil depths. Solid and striped columns represent 0-15 and 15-30 cm in depth, respectively. Two-way Analysis of Variance (ANOVA) with post hoc Tukey Honestly Significant Difference performed. Letters above bars indicant significant differences amongst treatments within their respective depth ($p < 0.05$); bars sharing the same letter are not significantly different. Errors bars represent standard deviation (±SD).

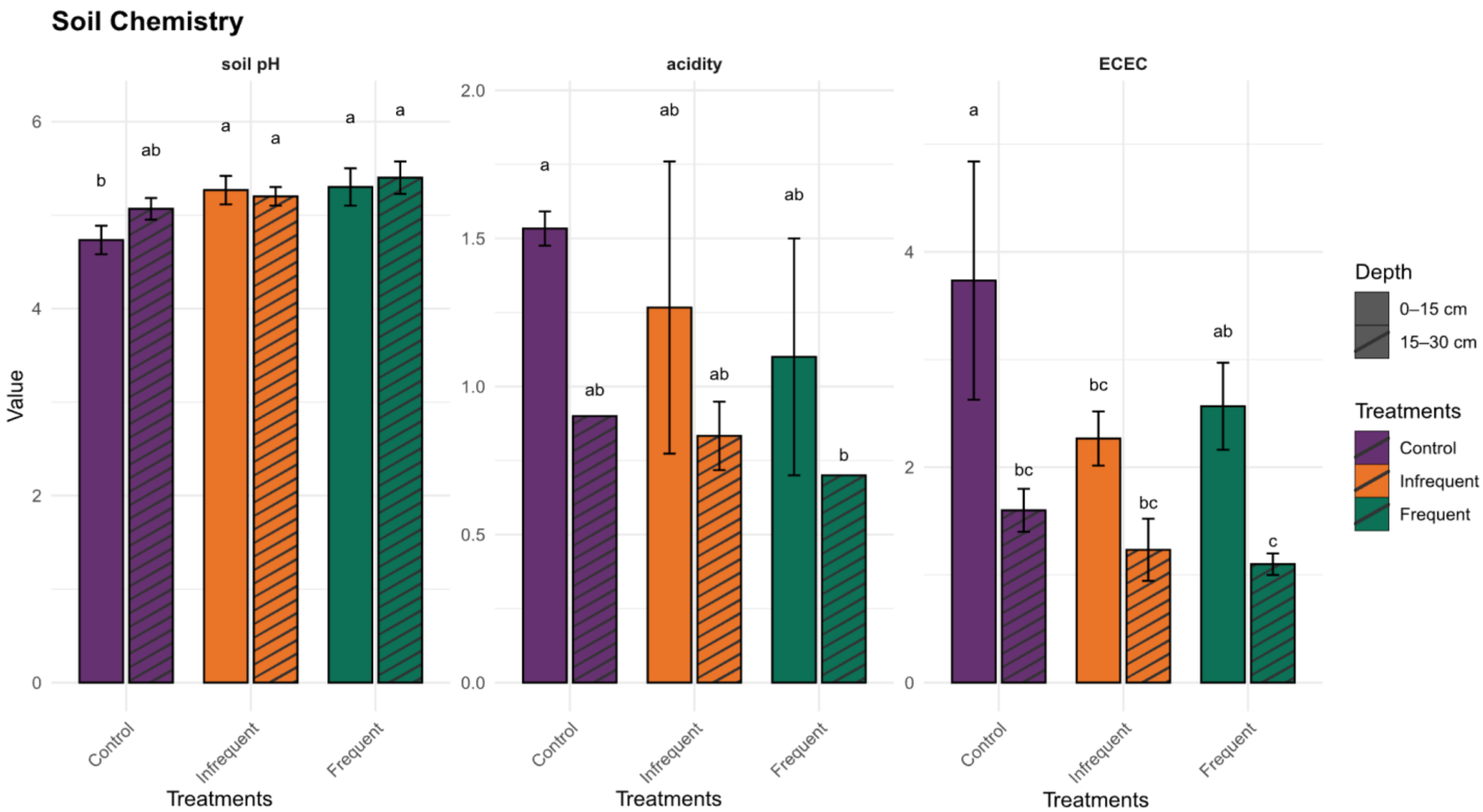

Figure S7: Averaged concentrations (±SD) of soil chemistry such as soil pH, acidity (*meq/100 g*), and effective cation exchange capacity (ECEC, *meg/100 g*) across all fire treatments and soil depths. Solid and striped columns represent 0-15 and 15-30 cm in depth, respectively. Two-way Analysis of Variance (ANOVA) with post hoc Tukey Honestly Significant Difference performed. Letters above bars indicant significant differences amongst treatments within their respective depth ($p < 0.05$); bars sharing the same letter are not significantly different. Errors bars represent standard deviation (±SD).

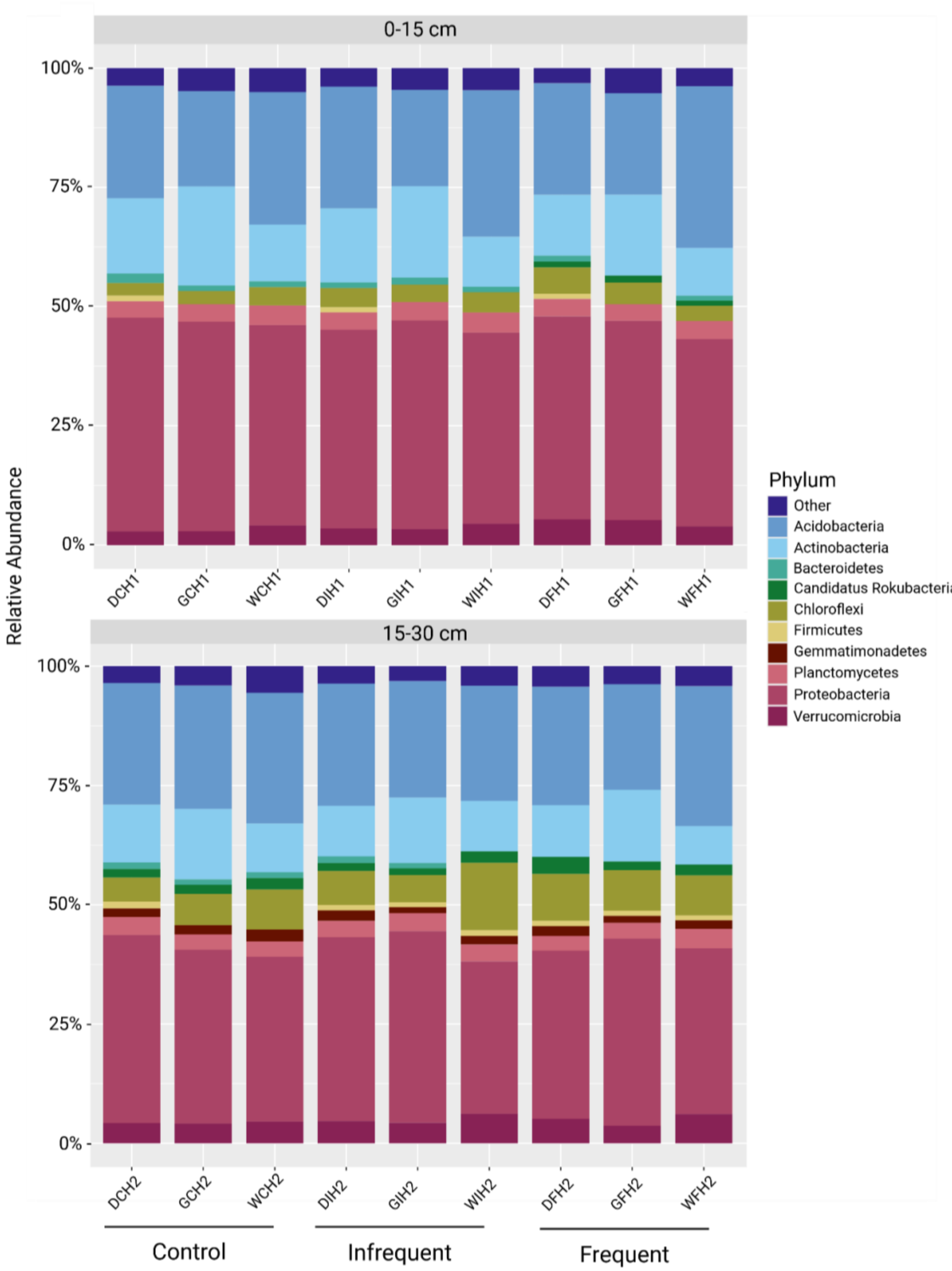

Figure S8. Relative abundance of prokaryotic communities at Phylum level across all samples. Community composition was determined by read-based taxonomic classification via Kaiju. Samples are grouped by treatment (Control, Infrequent, and Frequent) with shallower (0-15 cm) and deeper (15-30 cm) depths shown on the top and bottom, respectively. Colors correlate to relevant taxonomy with unclassified reads excluded and classified taxa comprising <0.01% relative abundance grouped into "other".

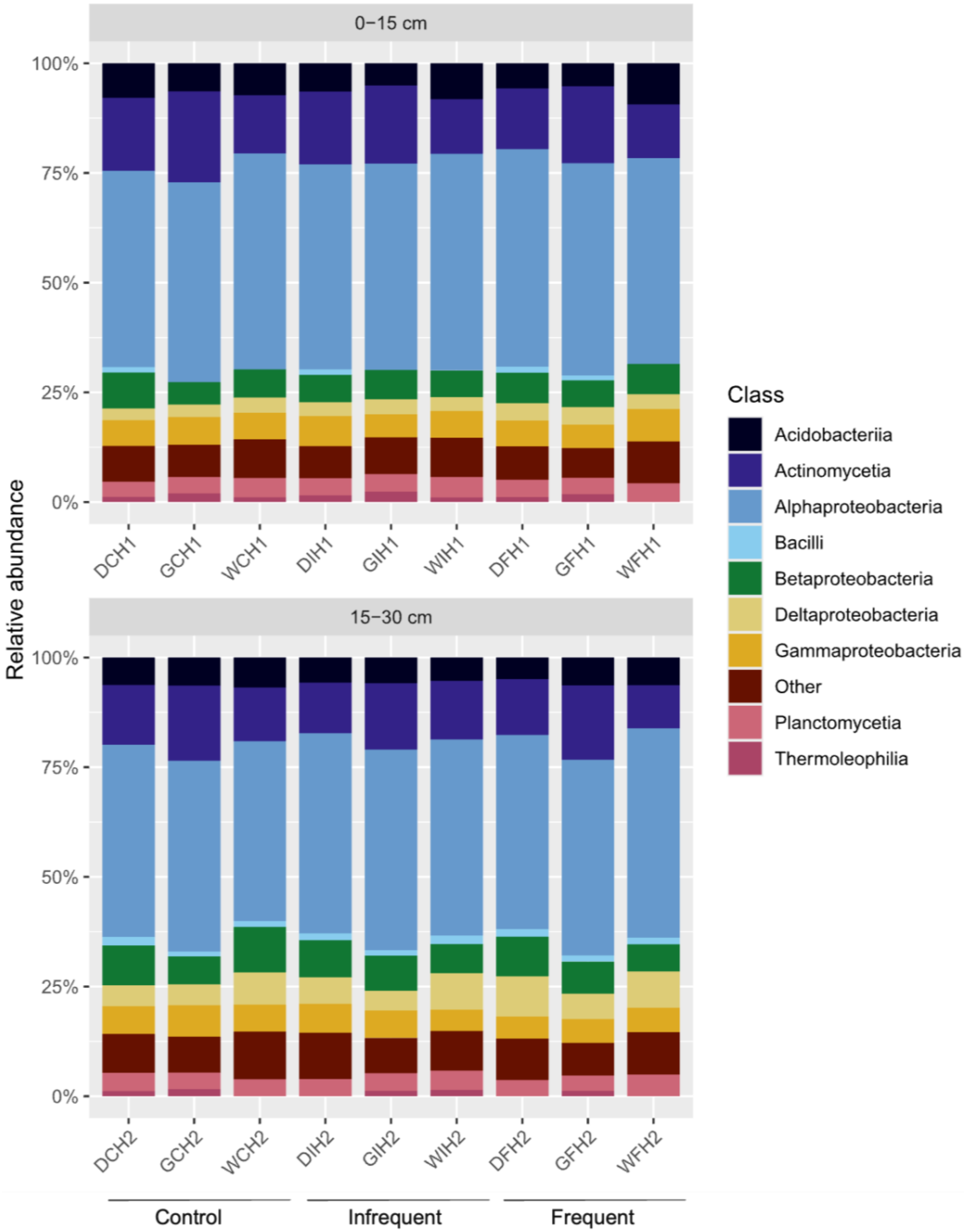

Figure S9. Relative abundance of prokaryotic communities at Class level across all samples. Community composition was determined by read-based taxonomic classification via Kaiju. Samples are grouped by treatment (Control, Infrequent, and Frequent) with shallower (0-15 cm) and deeper (15-30 cm) depths shown on the top and bottom, respectively. Colors correlate to relevant taxonomy with unclassified reads excluded and classified taxa comprising <0.01% relative abundance grouped into “other”.

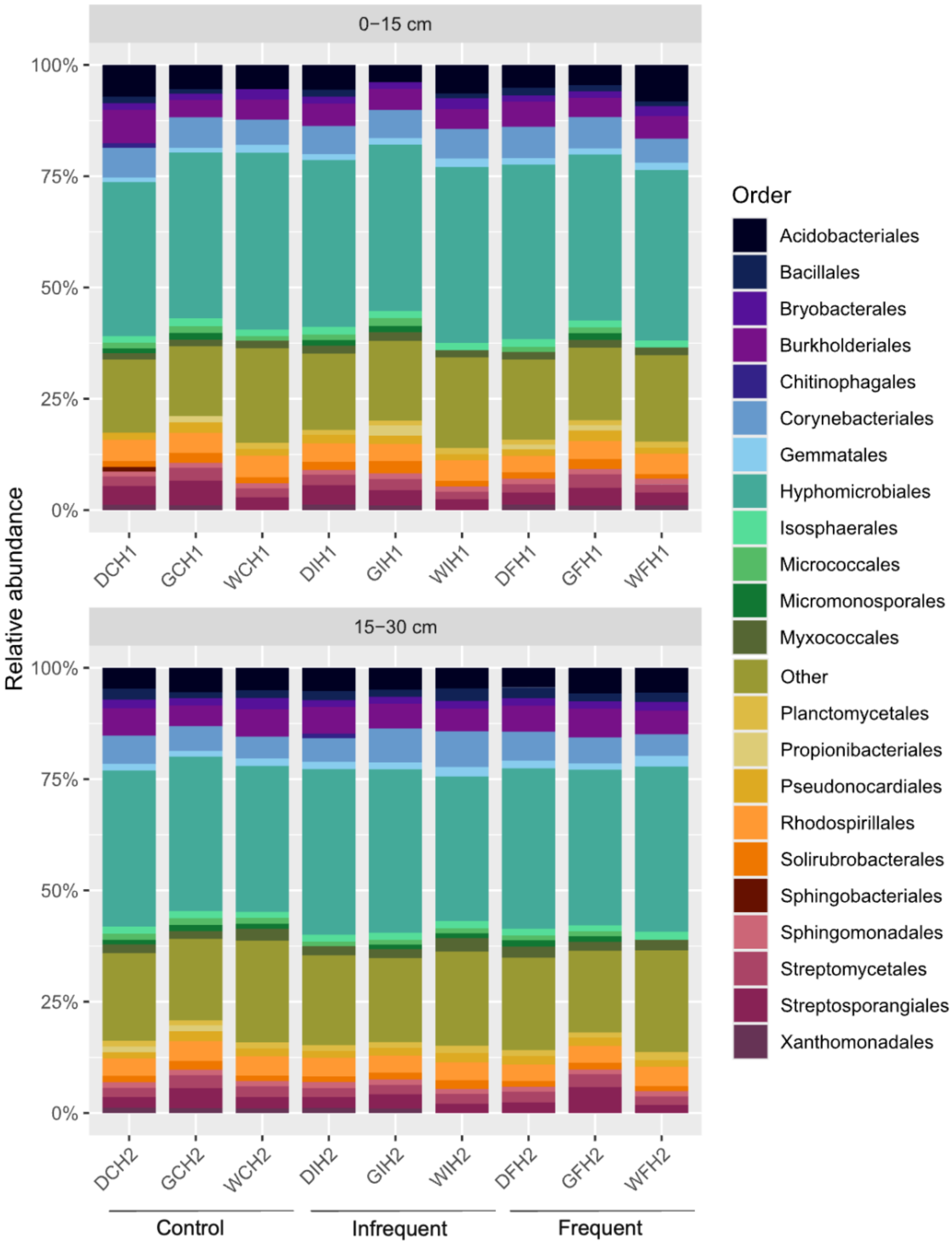

Figure S10: Relative abundance of prokaryotic communities at Order level across all samples. Community composition was determined by read-based taxonomic classification via Kaiju. Samples are grouped by treatment (Control, Infrequent, and Frequent) with shallower (0-15 cm) and deeper (15-30 cm) depths shown on the top and bottom, respectively. Colors correlate to relevant taxonomy with unclassified reads excluded and classified taxa comprising <0.01% relative abundance grouped into “other”.

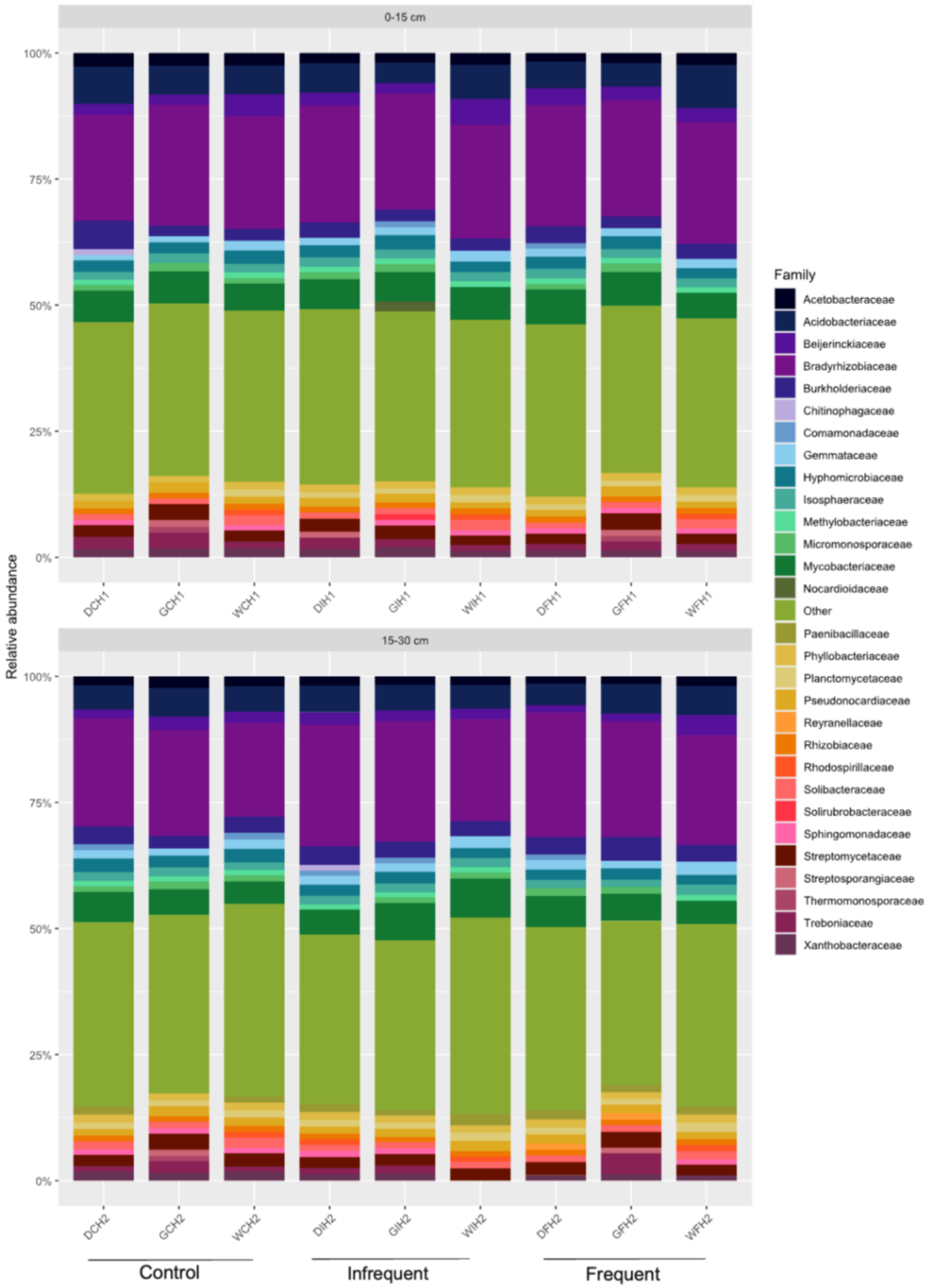

Figure S11: Relative abundance of prokaryotic communities at Family level across all samples. Community composition was determined by read-based taxonomic classification via Kaiju. Samples are grouped by treatment (Control, Infrequent, and Frequent) with shallower (0-15 cm) and deeper (15-30 cm) depths shown on the top and bottom, respectively. Colors correlate to relevant taxonomy with unclassified reads excluded and classified taxa comprising <0.01% relative abundance grouped into “other”.

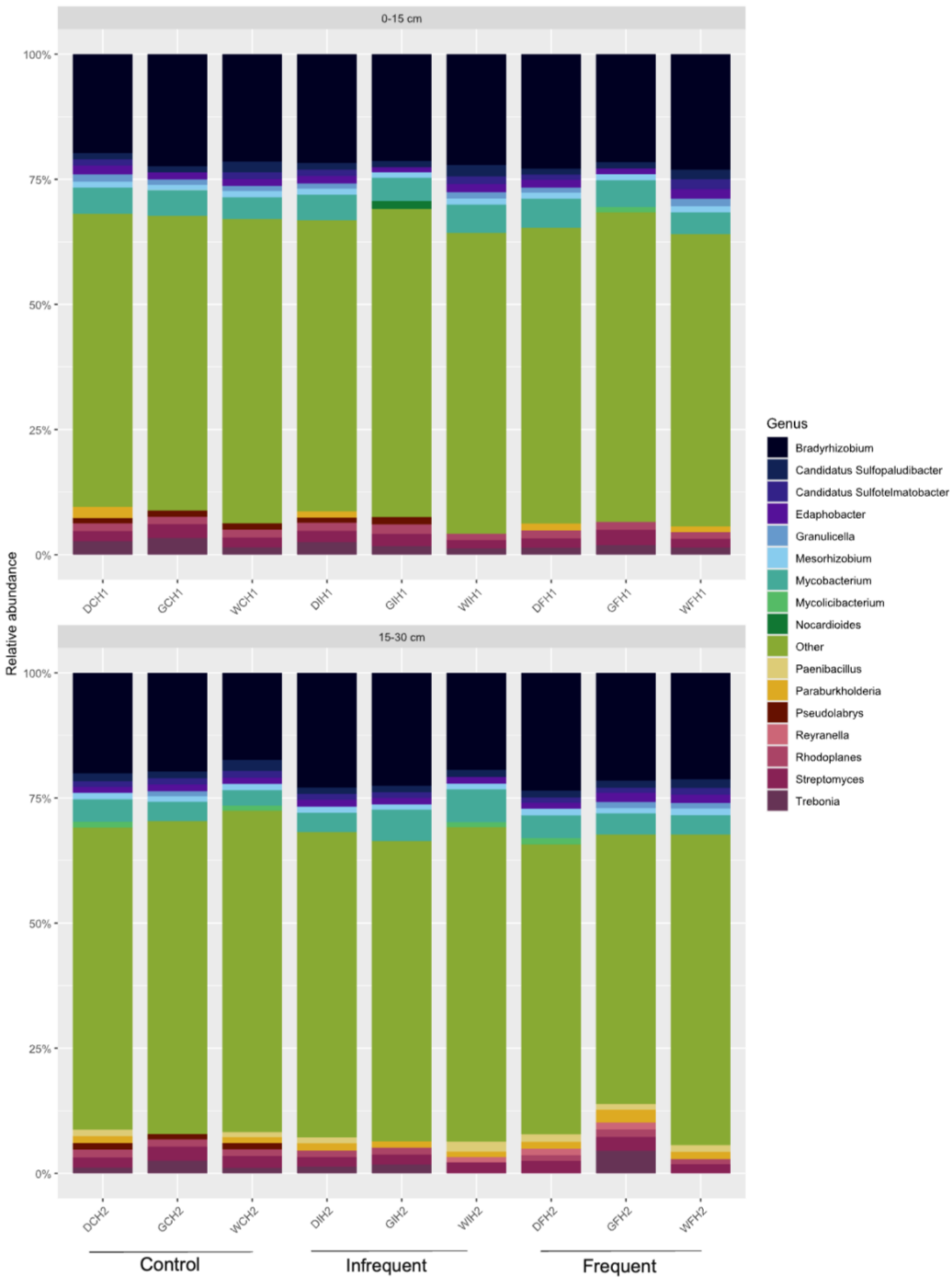

Figure S12: Relative abundance of prokaryotic communities at Genus level across all samples. Community composition was determined by read-based taxonomic classification via Kaiju. Samples are grouped by treatment (Control, Infrequent, and Frequent) with shallower (0-15 cm) and deeper (15-30 cm) depths shown on the top and bottom, respectively. Colors correlate to relevant taxonomy with unclassified reads excluded and classified taxa comprising <0.01% relative abundance grouped into “other”.

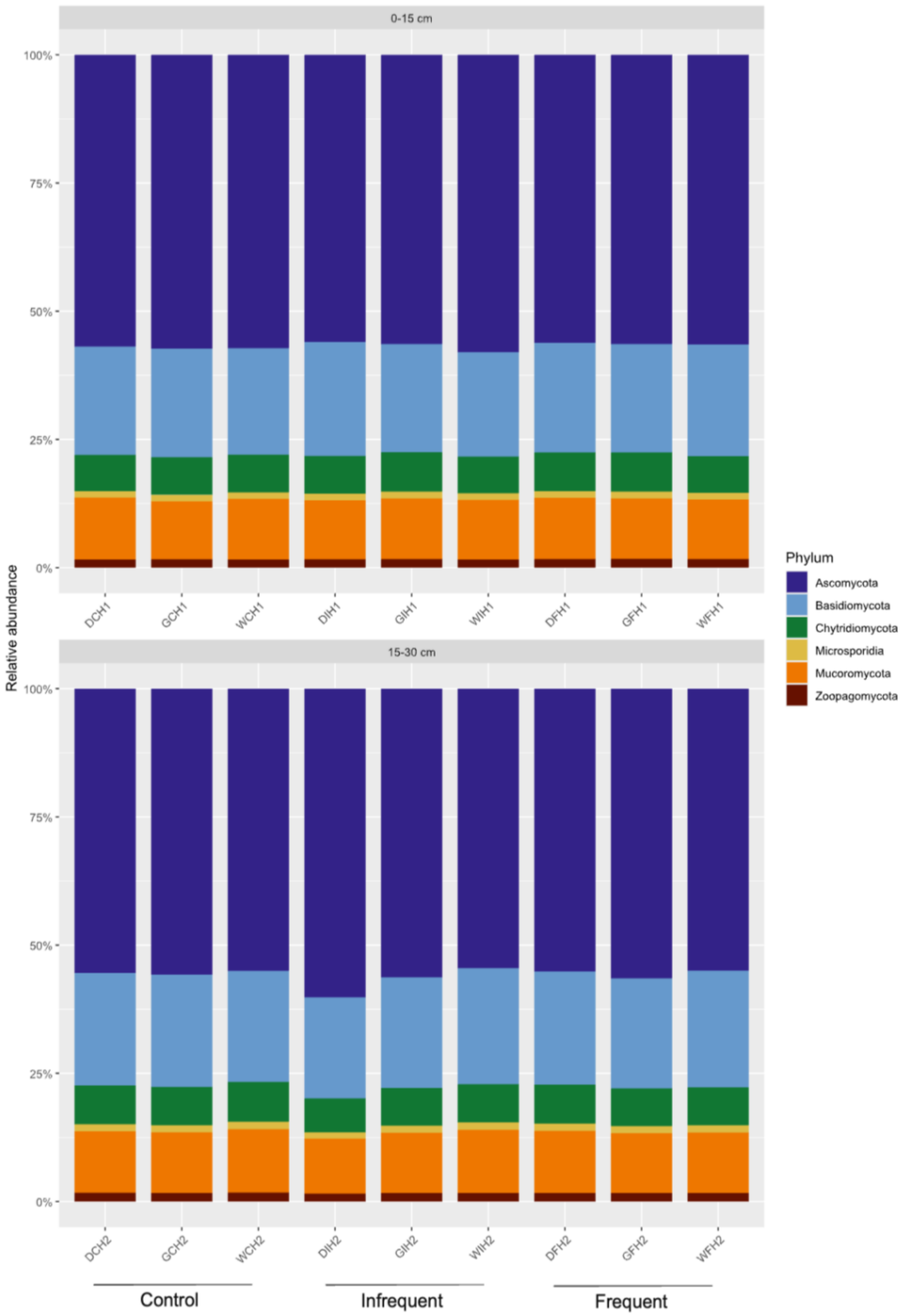

Figure S13: Relative abundance of fungal communities at Phylum level across all samples. Community composition was determined by read-based taxonomic classification via Kaiju. Samples are grouped by treatment (Control, Infrequent, and Frequent) with shallower (0-15 cm) and deeper (15-30 cm) depths shown on the top and bottom, respectively. Colors correlate to relevant taxonomy with unclassified reads excluded and classified taxa comprising <0.01% relative abundance grouped into “other”.

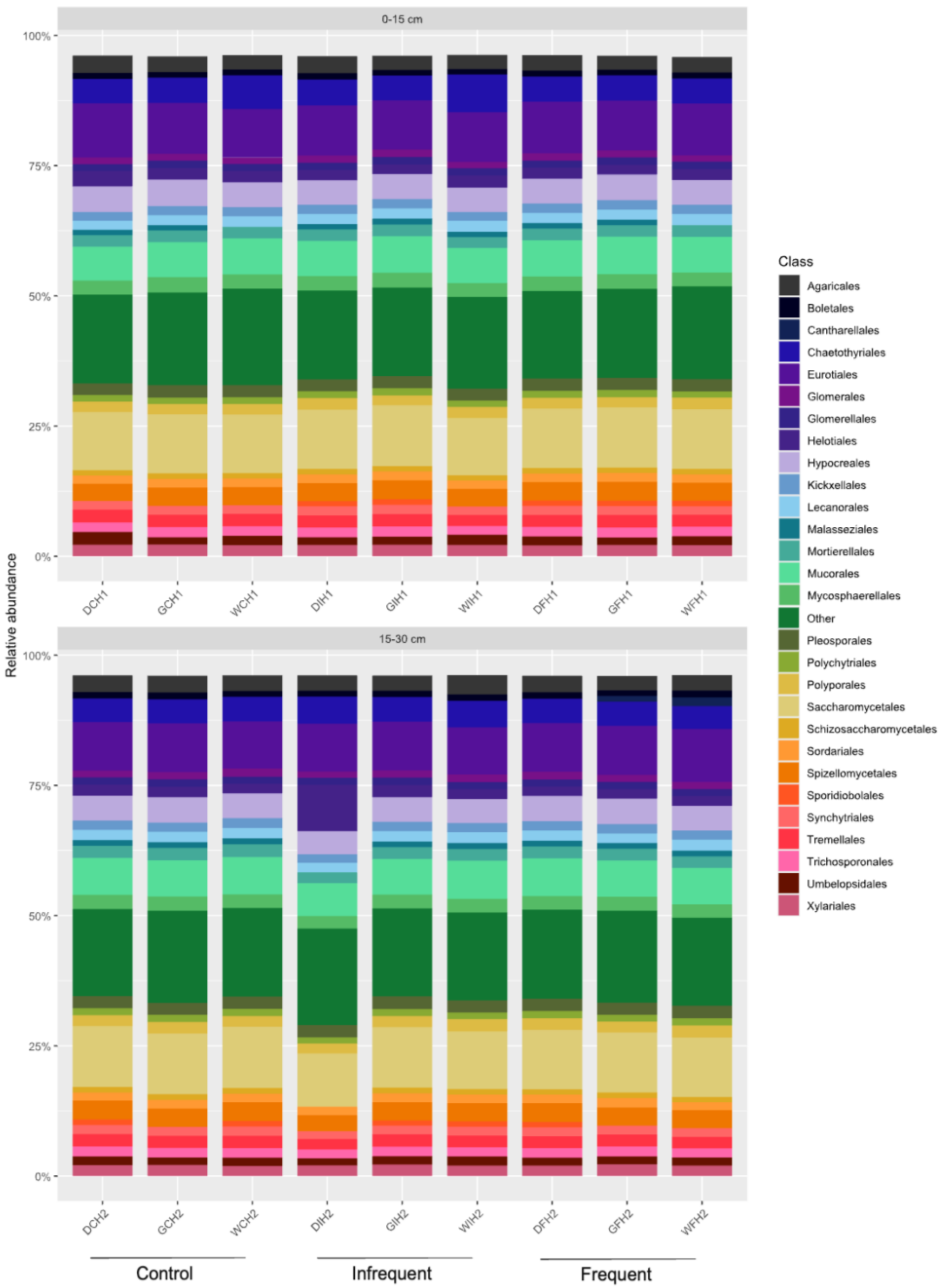

Figure S14: Relative abundance of fungal communities at Class level across all samples. Community composition was determined by read-based taxonomic classification via Kaiju. Samples are grouped by treatment (Control, Infrequent, and Frequent) with shallower (0-15 cm) and deeper (15-30 cm) depths shown on the top and bottom, respectively. Colors correlate to relevant taxonomy with unclassified reads excluded and classified taxa comprising <0.01% relative abundance grouped into “other”.

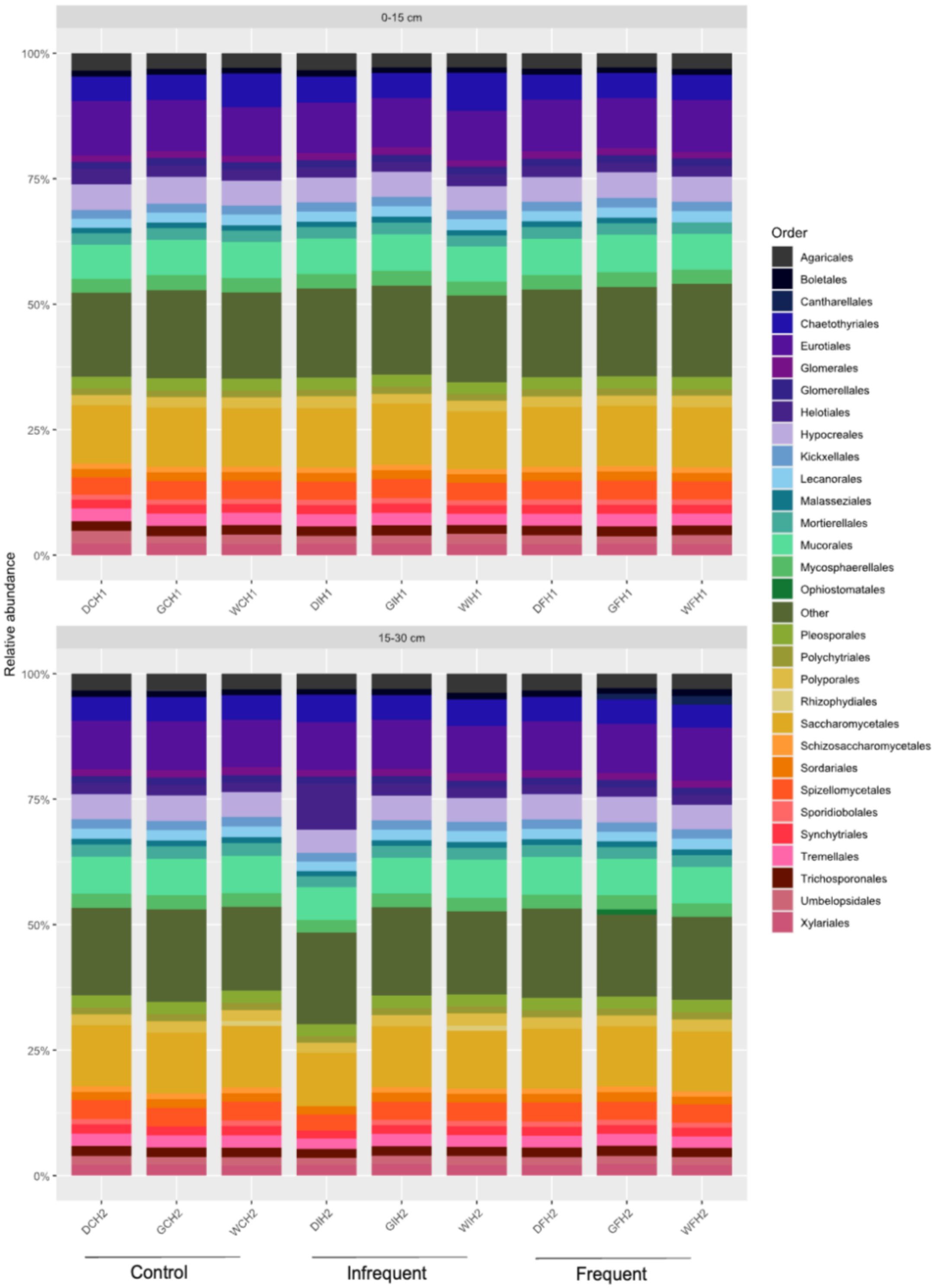

Figure S15: Relative abundance of fungal communities at Order level across all samples. Community composition was determined by read-based taxonomic classification via Kaiju. Samples are grouped by treatment (Control, Infrequent, and Frequent) with shallower (0-15 cm) and deeper (15-30 cm) depths shown on the top and bottom, respectively. Colors correlate to relevant taxonomy with unclassified reads excluded and classified taxa comprising <0.01% relative abundance grouped into “other”.

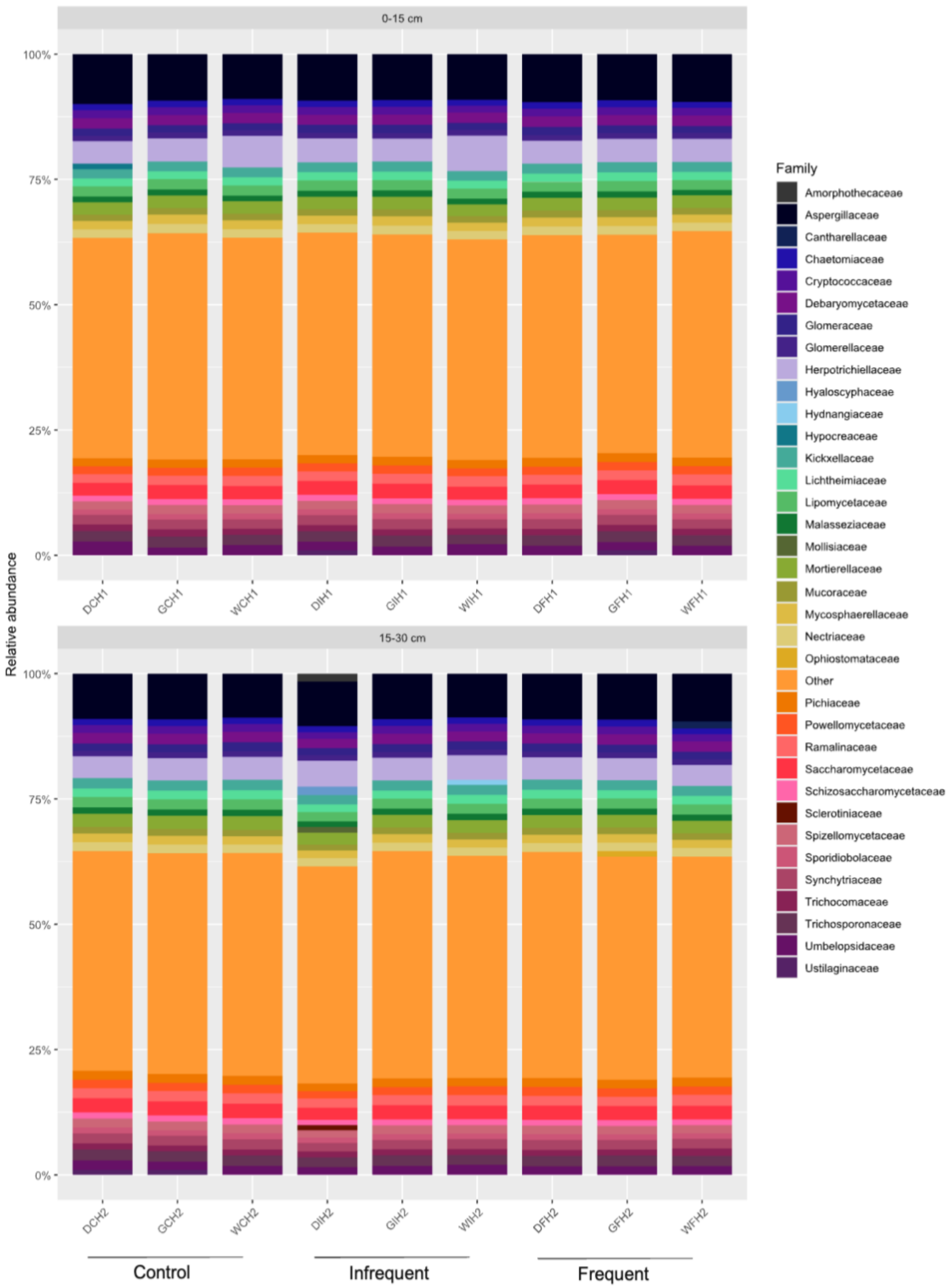

Figure S16: Relative abundance of fungal communities at Family level across all samples. Community composition was determined by read-based taxonomic classification via Kaiju. Samples are grouped by treatment (Control, Infrequent, and Frequent) with shallower (0-15 cm) and deeper (15-30 cm) depths shown on the top and bottom, respectively. Colors correlate to relevant taxonomy with unclassified reads excluded and classified taxa comprising <0.01% relative abundance grouped into “other”.

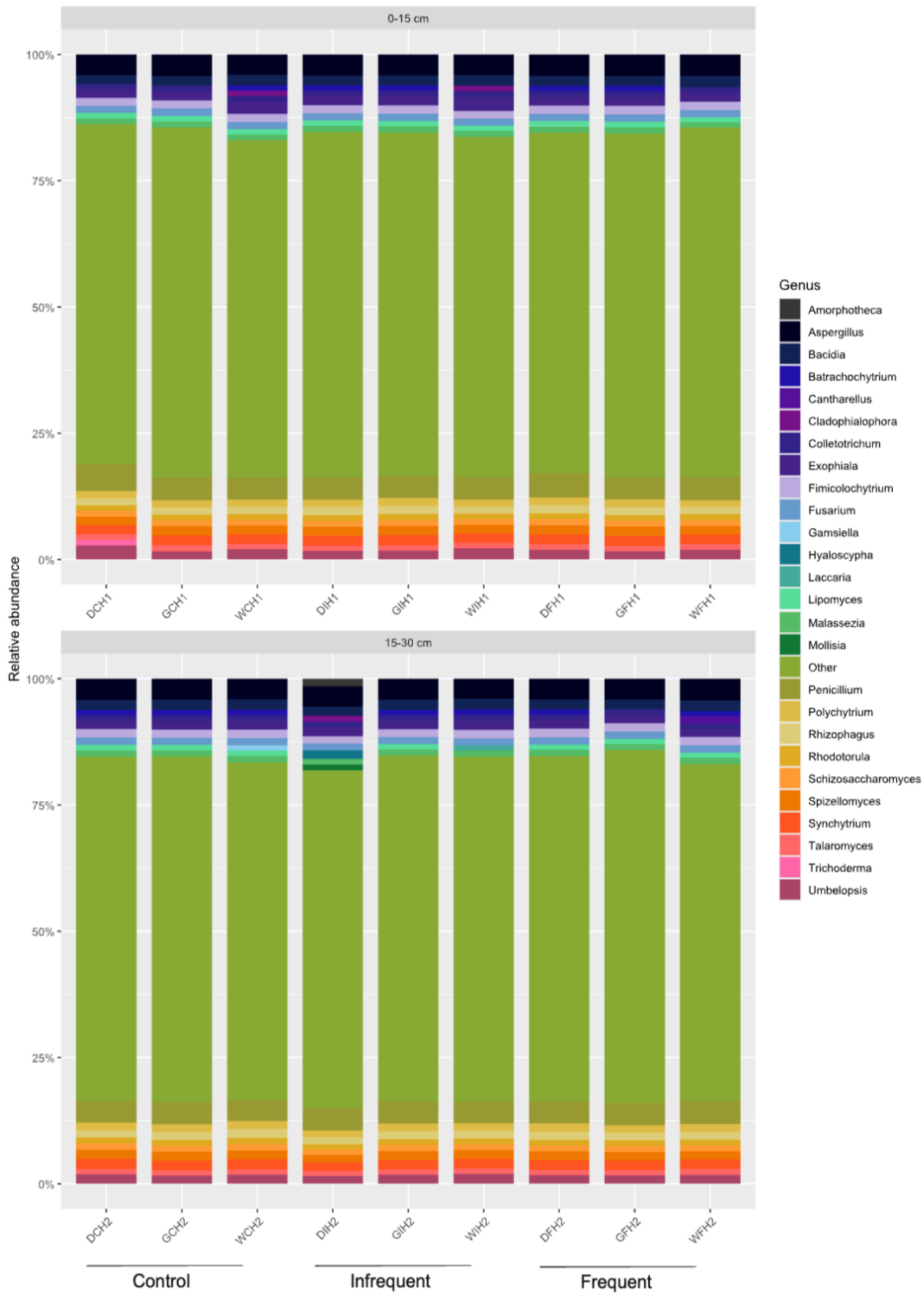

Figure S17: Relative abundance of fungal communities at Genus level across all samples. Community composition was determined by read-based taxonomic classification via Kaiju. Samples are grouped by treatment (Control, Infrequent, and Frequent) with shallower (0-15 cm) and deeper (15-30 cm) depths shown on the top and bottom, respectively. Colors correlate to relevant taxonomy with unclassified reads excluded and classified taxa comprising <0.01% relative abundance grouped into “other”.

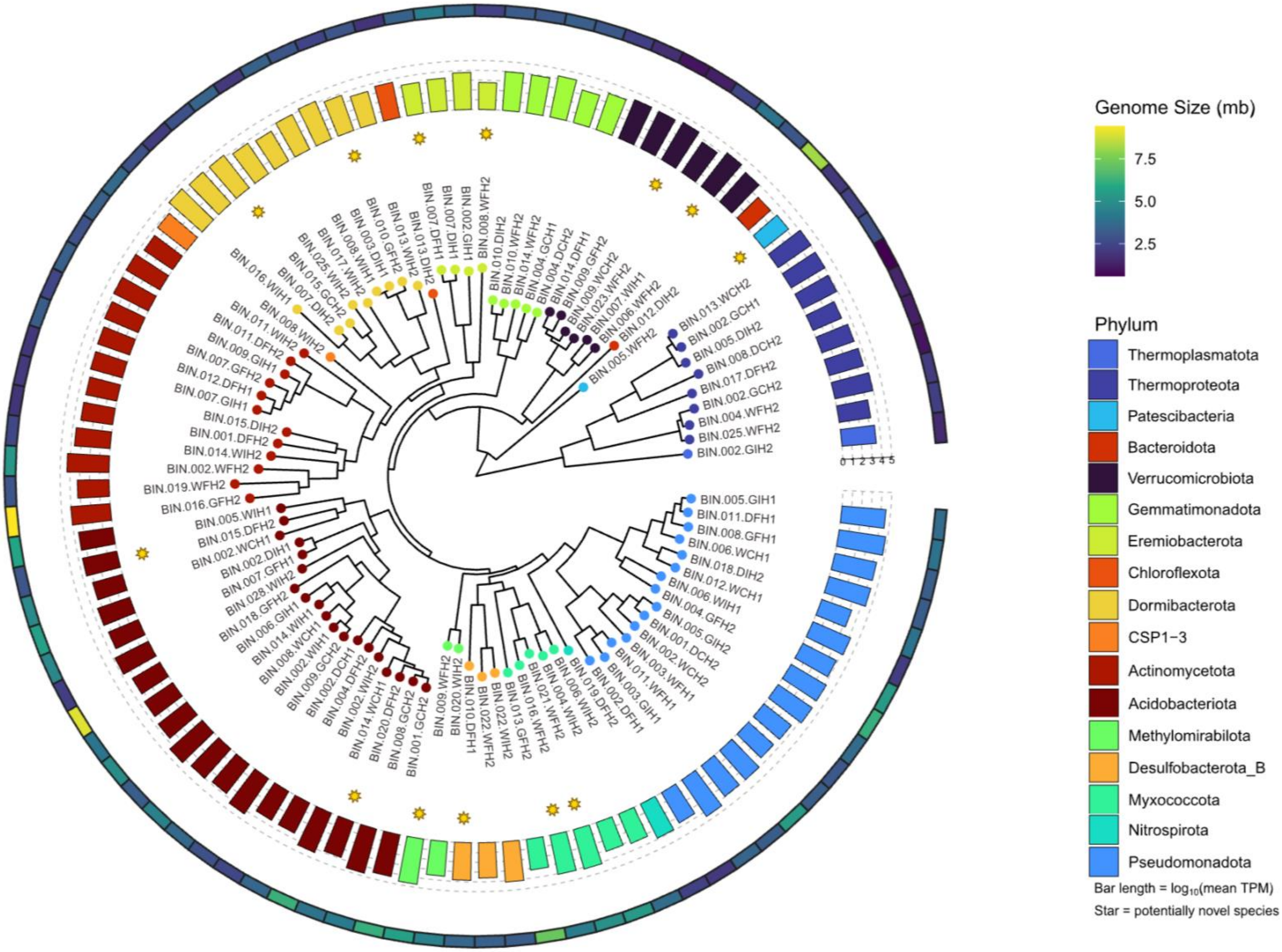

Figure S18: Approximately maximum-likelihood phylogenetic tree (using FastTree2) of MAGs from the Albany Pine Bush Preserve colored by phyla, with branches proportional to genetic distances between MAGs. From outermost ring to center: Outermost ring (colored tiles) displays average genome size in megabase pairs (Mb), scale shown on right, (lighter = larger genomes, darker = smaller genomes), ranging from approximately 0.5 to 9.5 Mb. Second outermost ring (bar chart) represents $\log_{10}$ mean transcripts per million (TPM) of each bin. Longer bars correspond to higher TPM. Third ring (gold stars) indicate potentially novel species. Center (phylogenic tree) represents the phylogenetic relationship amongst recovered MAGs, labeled "BIN.xxx.xxxx", with D/G/W = site, C/I/F = fire treatment, H1/2 = soil depth.

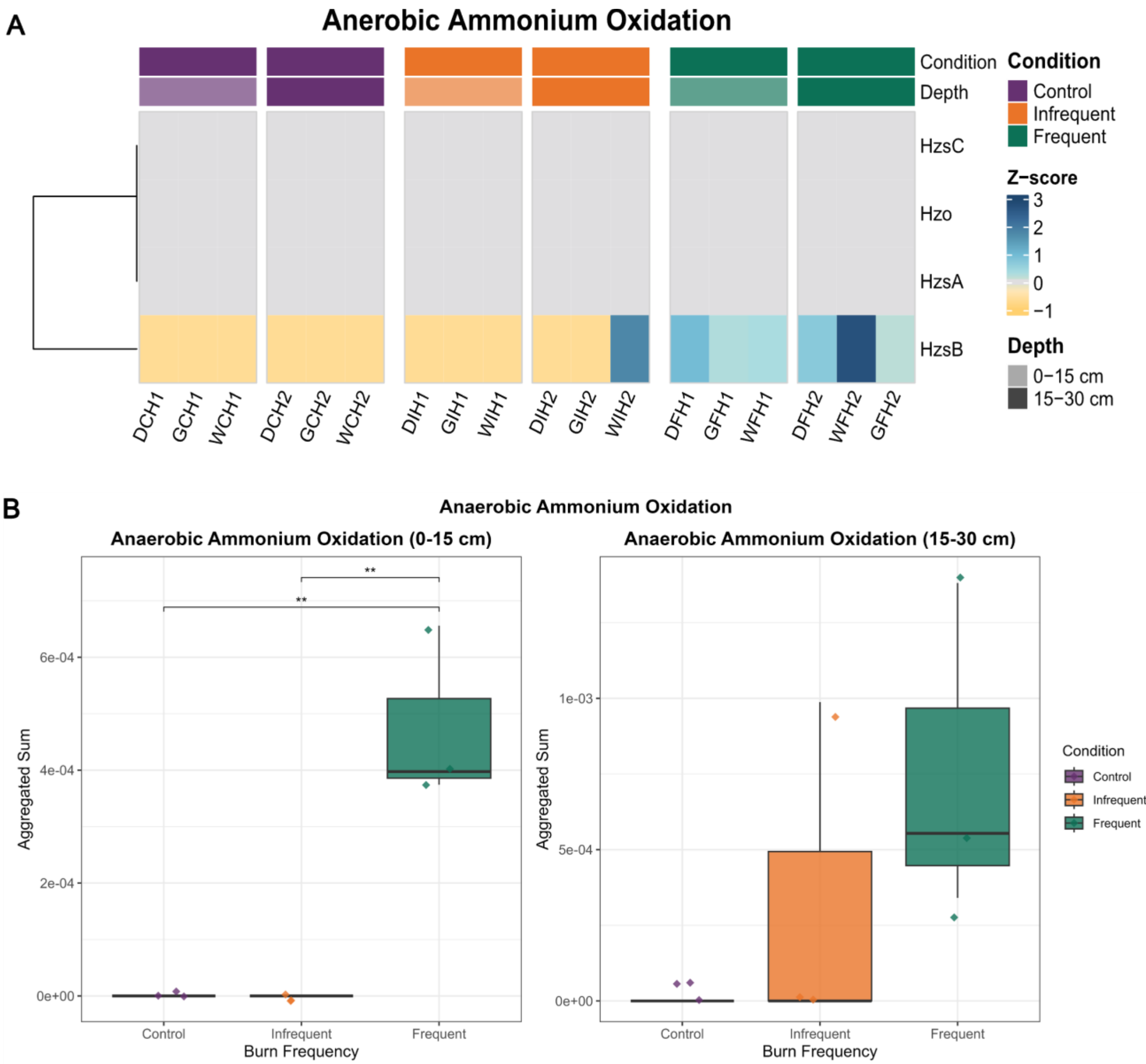

Figure S19. A) Normalized heatmap of gene present within the anaerobic ammonium oxidation (NH_4^+ + NO_2^- → N_2) pathway. Each row represents an individual gene and each column represents a sample, grouped by burn frequency treatment (Control, Infrequent, Frequent) and soil depth (0–15 cm, lighter shading; 15–30 cm, darker shading). Gene abundance values were normalized by Z-score across samples, such that each value reflects the number of standard deviations above or below the mean abundance for that gene across all samples. Blue indicates above-average abundance (positive Z-score), yellow-orange indicates below-average abundance (negative Z-score), and grey cells indicate an abundance of zero. Completely greyed-out rows indicate genes for which no data were available across any sample. **B)** box-and-whisker plot of the sum of all genes (in EFPKG) in the anaerobic ammonia oxidation pathway from each site at both soil depths. Significance was determined using a one-way ANOVA with a Tukey HSD post hoc test. Adjusted p-values were used to determine significance (*p.adj < 0.05*): * = *p.adj <0.05, ** = p.adj <0.01, *** = p.adj <0.001*, no stars = no significance. Details on the genes grouped in each pathway are shown in **Table S4** while the pathways themselves are visualized in **Figure S2.**

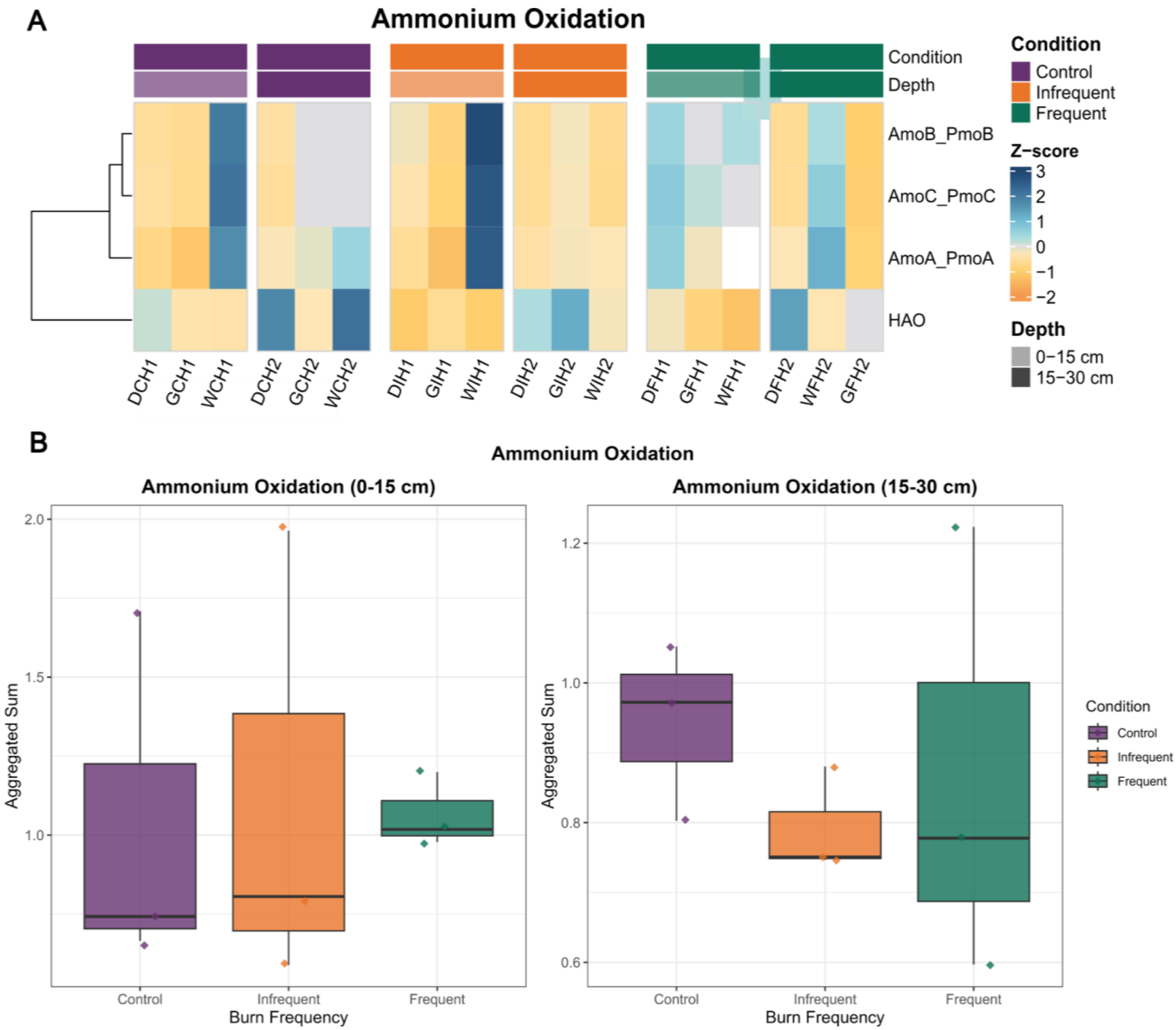

Figure S20. A) Normalized heatmap of gene present within the ammonia oxidation pathway ($NH_4^+ \rightarrow NO_2^{2-}$). Each row represents an individual gene and each column represents a sample, grouped by burn frequency treatment (Control, Infrequent, Frequent) and soil depth (0–15 cm, lighter shading; 15–30 cm, darker shading). Gene abundance values were normalized by Z-score across samples, such that each value reflects the number of standard deviations above or below the mean abundance for that gene across all samples. Blue indicates above-average abundance (positive Z-score), yellow-orange indicates below-average abundance (negative Z-score), and grey cells indicate an abundance of zero. Completely greyed-out rows indicate genes for which no data were available across any sample. **B)** box-and-whisker plot of the sum of all genes (in EFPKG) in the ammonium oxidation pathway from each site at both soil depths. Significance was determined using a one-way ANOVA with a Tukey HSD post hoc test. Adjusted p-values were used to determine significance (*p.adj < 0.05*): * = *p.adj <0.05,* ** = *p.adj <0.01,* *** = *p.adj <0.001*, no stars = no significance*.* Details on the genes grouped in each pathway are shown in **Table S4** while the pathways themselves are visualized in **Figure S2.**

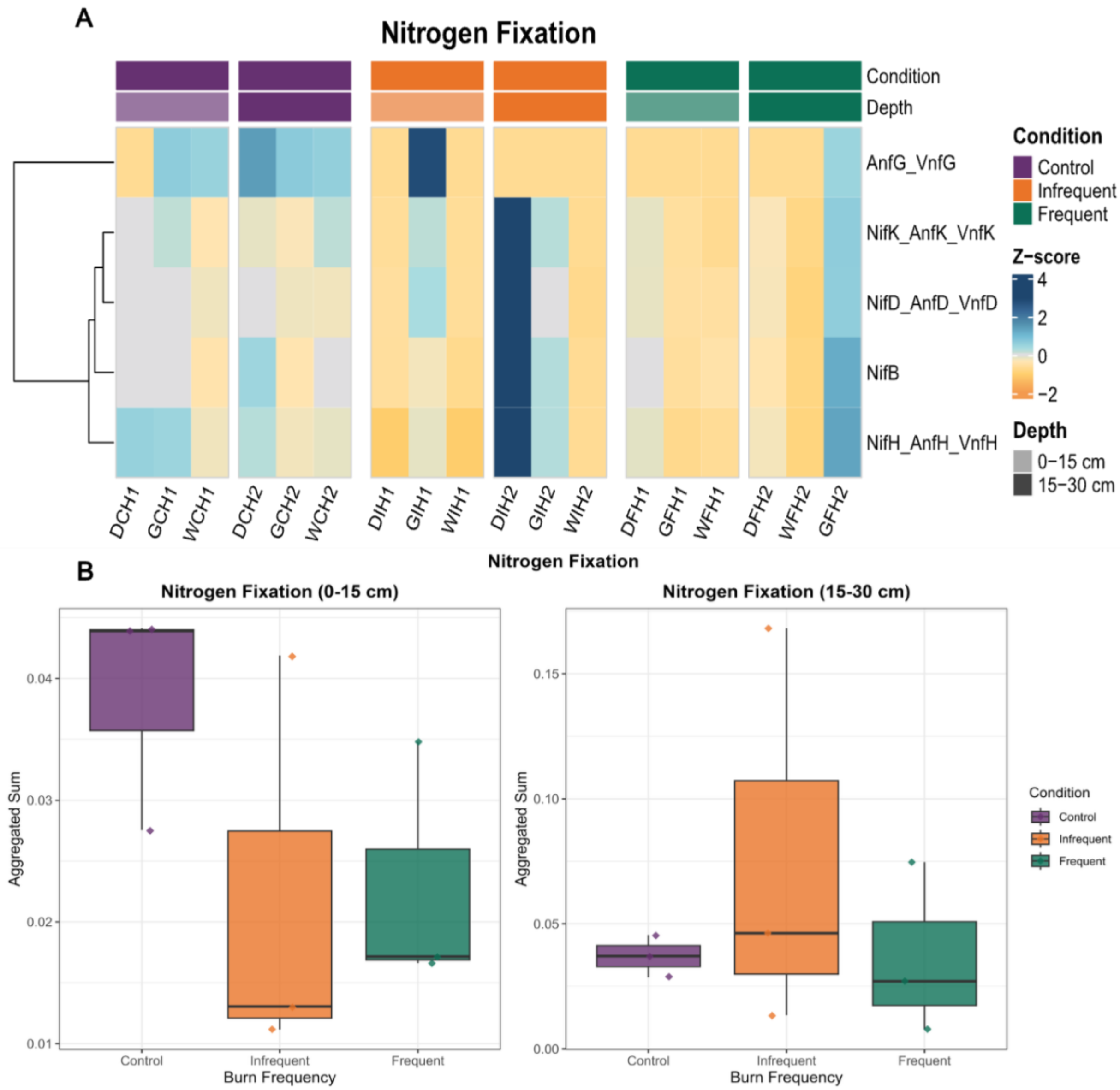

Figure S21. A) Normalized heatmap of gene present within the nitrogen fixation pathway ($N_2 \rightarrow NH_4^+$). Each row represents an individual gene and each column represents a sample, grouped by burn frequency treatment (Control, Infrequent, Frequent) and soil depth (0–15 cm, lighter shading; 15–30 cm, darker shading). Gene abundance values were normalized by Z-score across samples, such that each value reflects the number of standard deviations above or below the mean abundance for that gene across all samples. Blue indicates above-average abundance (positive Z-score), yellow-orange indicates below-average abundance (negative Z-score), and grey cells indicate an abundance of zero. Completely greyed-out rows indicate genes for which no data were available across any sample. **B)** box-and-whisker plot of the sum of all genes (in EFPKG) in the nitrogen fixation pathway from each site at both soil depths. Significance was determined using a one-way ANOVA with a Tukey HSD post hoc test. Adjusted p-values were used to determine significance (*p.adj < 0.05*): * = *p.adj <0.05,* ** = *p.adj <0.01,* *** = *p.adj <0.001*, no stars = no significance*.* Details on the genes grouped in each pathway are shown in **Table S4** while the pathways themselves are visualized in **Figure S2.**

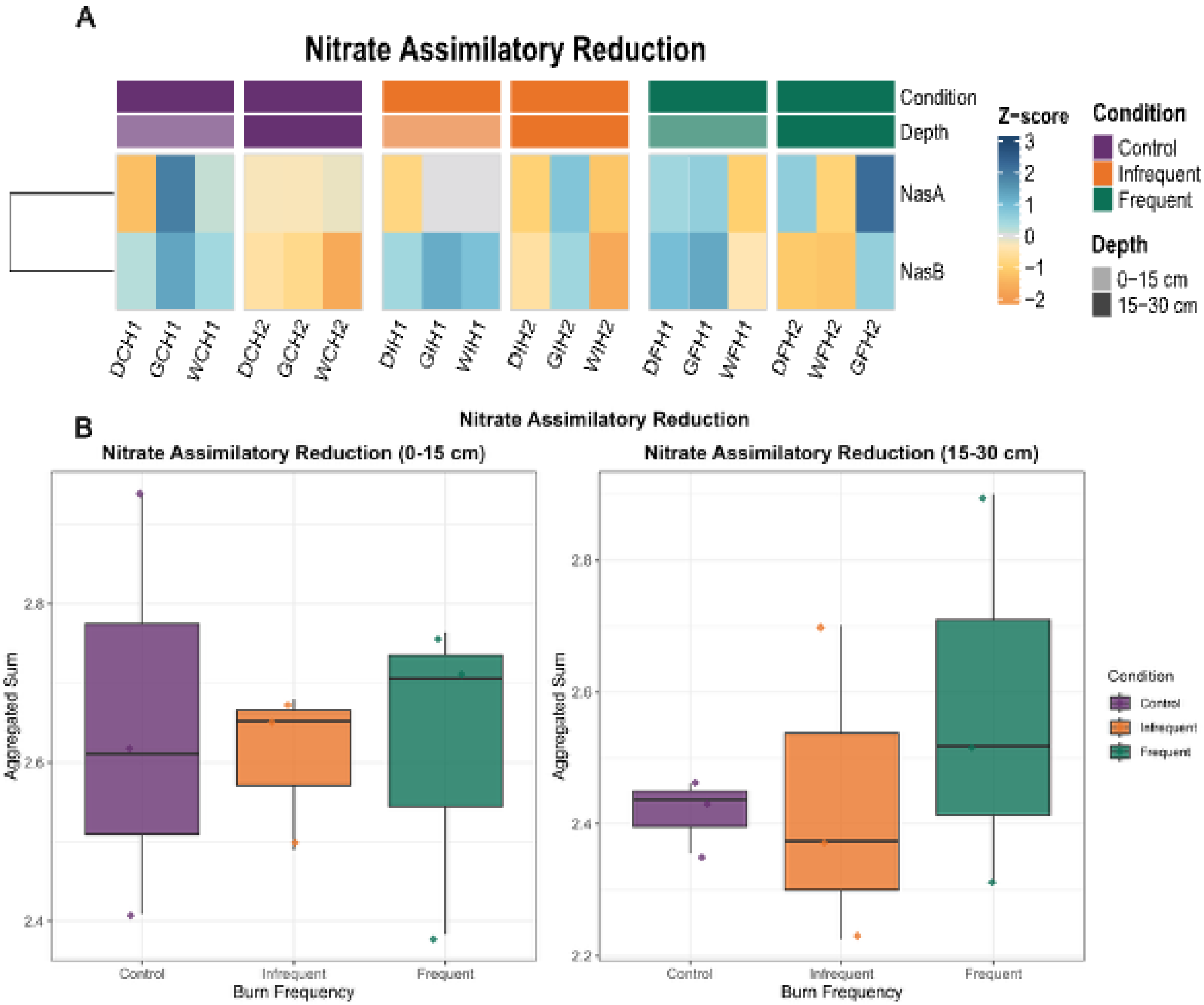

Figure S22. A) Normalized heatmap of gene present within the assimilatory nitrate reduction pathway (NO_3^{2-} → NO_2^{2-}). Each row represents an individual gene and each column represents a sample, grouped by burn frequency treatment (Control, Infrequent, Frequent) and soil depth (0–15 cm, lighter shading; 15–30 cm, darker shading). Gene abundance values were normalized by Z-score across samples, such that each value reflects the number of standard deviations above or below the mean abundance for that gene across all samples. Blue indicates above-average abundance (positive Z-score), yellow-orange indicates below-average abundance (negative Z-score), and grey cells indicate an abundance of zero. Completely greyed-out rows indicate genes for which no data were available across any sample. **B)** box-and-whisker plot of the sum of all genes (in EFPKG) in the assimilatory nitrate reduction pathway from each site at both soil depths. Significance was determined using a one-way ANOVA with a Tukey HSD post hoc test. Adjusted p-values were used to determine significance (*p.adj < 0.05*): * = *p.adj <0.05,* ** = *p.adj <0.01,* *** = *p.adj <0.001*, no stars = no significance*.* Details on the genes grouped in each pathway are shown in **Table S4** while the pathways themselves are visualized in **Figure S2.**

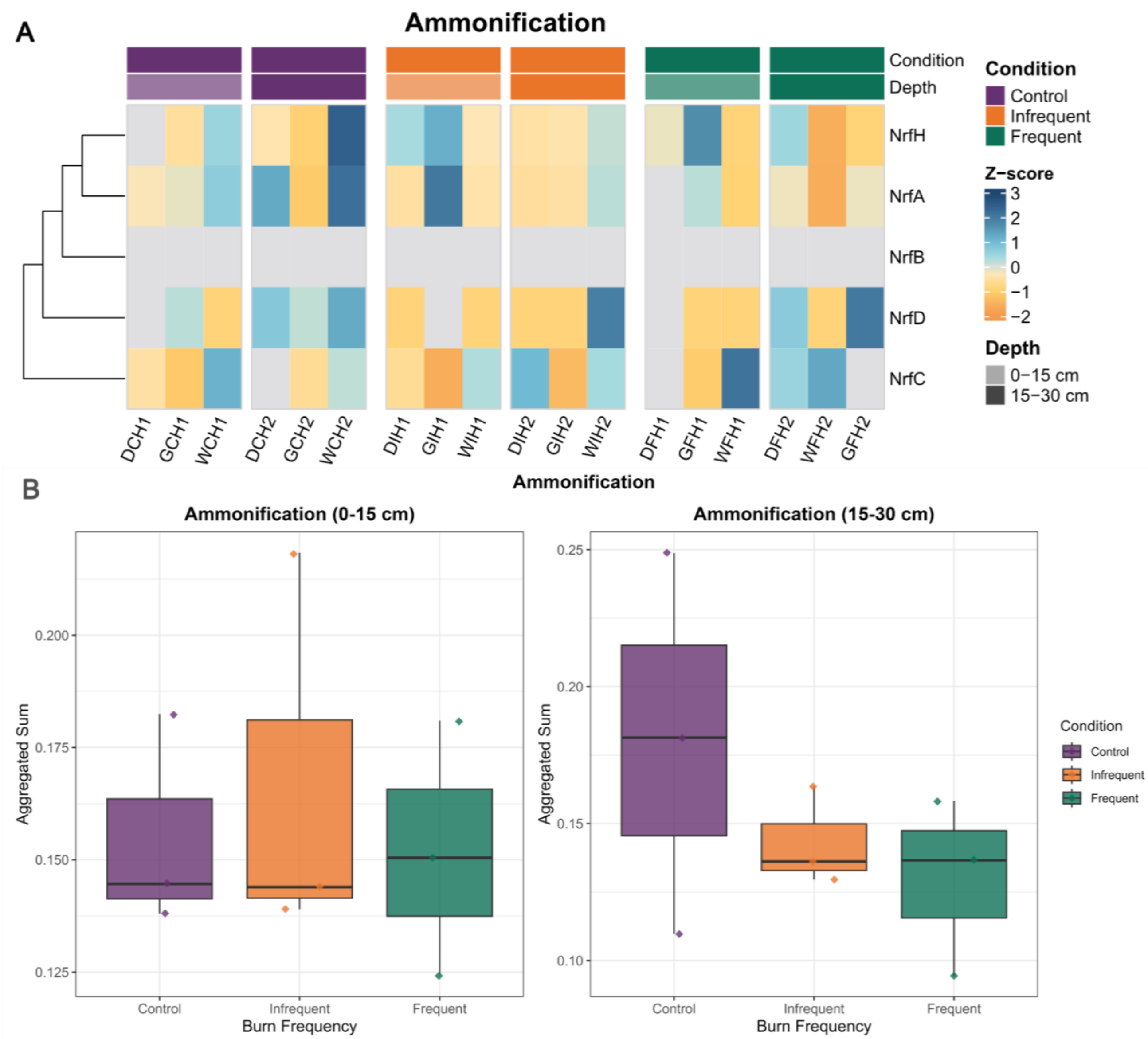

Figure S23. A) Normalized heatmap of gene present within the ammonification pathway (NO_2^- → NH_4^+). Each row represents an individual gene and each column represents a sample, grouped by burn frequency treatment (Control, Infrequent, Frequent) and soil depth (0–15 cm, lighter shading; 15–30 cm, darker shading). Gene abundance values were normalized by Z-score across samples, such that each value reflects the number of standard deviations above or below the mean abundance for that gene across all samples. Blue indicates above-average abundance (positive Z-score), yellow-orange indicates below-average abundance (negative Z-score), and grey cells indicate an abundance of zero. Completely greyed-out rows indicate genes for which no data were available across any sample. **B)** box-and-whisker plot of all genes in ammonification pathway from each site in both soil depth (0-15 cm) and (15-30 cm) respectively. Gene data were aggregated and clustered by pathway. ANOVA Tukey post hoc statisical signifance signified: * = *p <0.05, ** = p <0.01, *** = p <0.001*, no stars = no significance. Details on the genes grouped in each pathway are shown in **Table S4** while the pathways themselves are visualized in **Figure S2.**

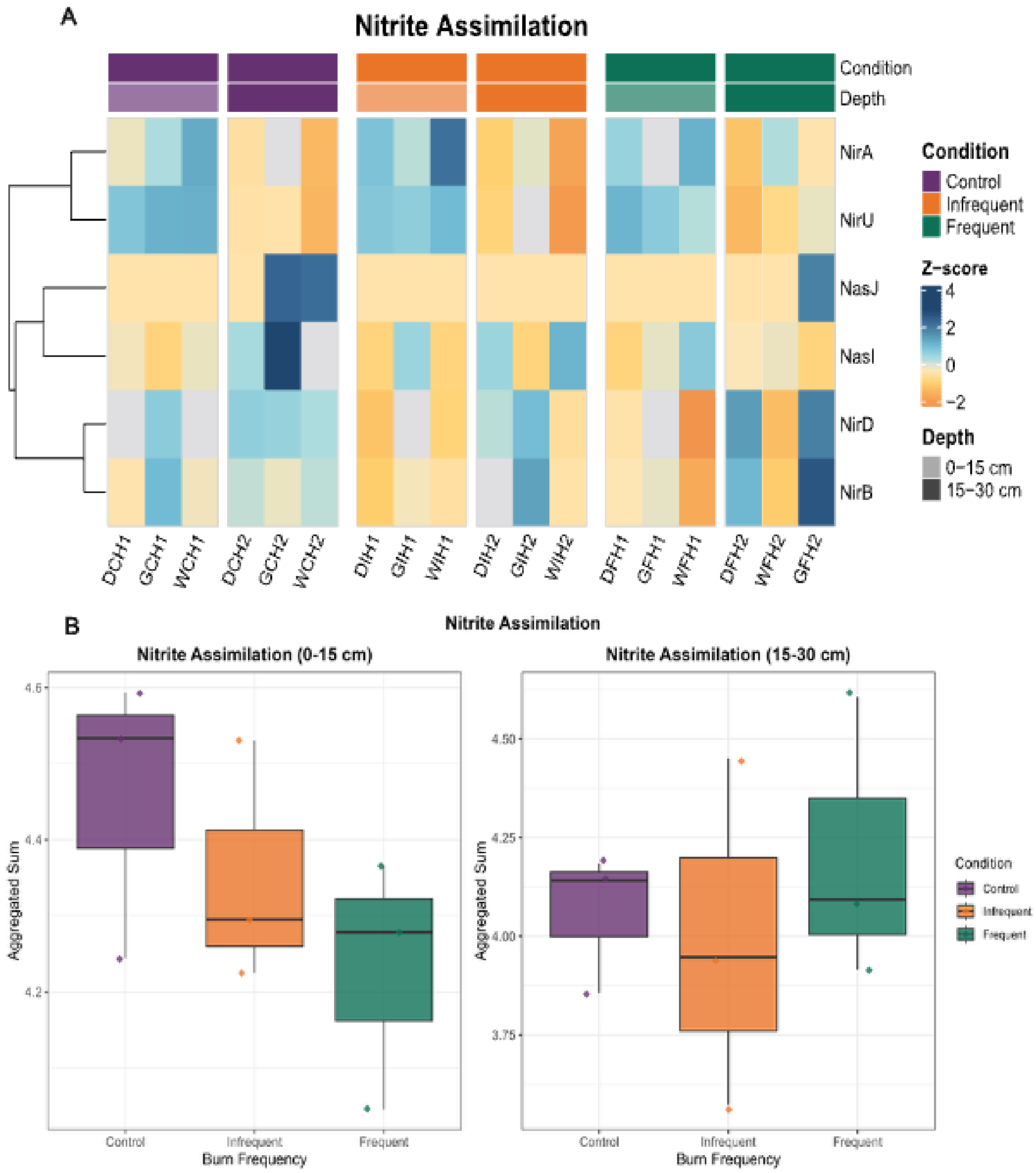

Figure S24. A) Normalized heatmap of gene present within the nitrite assimilation pathway (NO_2^{2-} → NH_4^+). Each row represents an individual gene and each column represents a sample, grouped by burn frequency treatment (Control, Infrequent, Frequent) and soil depth (0–15 cm, lighter shading; 15–30 cm, darker shading). Gene abundance values were normalized by Z-score across samples, such that each value reflects the number of standard deviations above or below the mean abundance for that gene across all samples. Blue indicates above-average abundance (positive Z-score), yellow-orange indicates below-average abundance (negative Z-score), and grey cells indicate an abundance of zero. Completely greyed-out rows indicate genes for which no data were available across any sample. **B)** box-and-whisker plot of all genes in nitrite assimilation pathway from each site in both soil depth (0-15 cm) and (15-30 cm) respectively. Gene data were aggregated and clustered by pathway. ANOVA Tukey post hoc statisical signifance signified: * = *p <0.05,* ** = *p <0.01,* *** = *p <0.001*, no stars = no significance. Details on the genes grouped in each pathway are shown in **Table S4** while the pathways themselves are visualized in **Figure S2.**

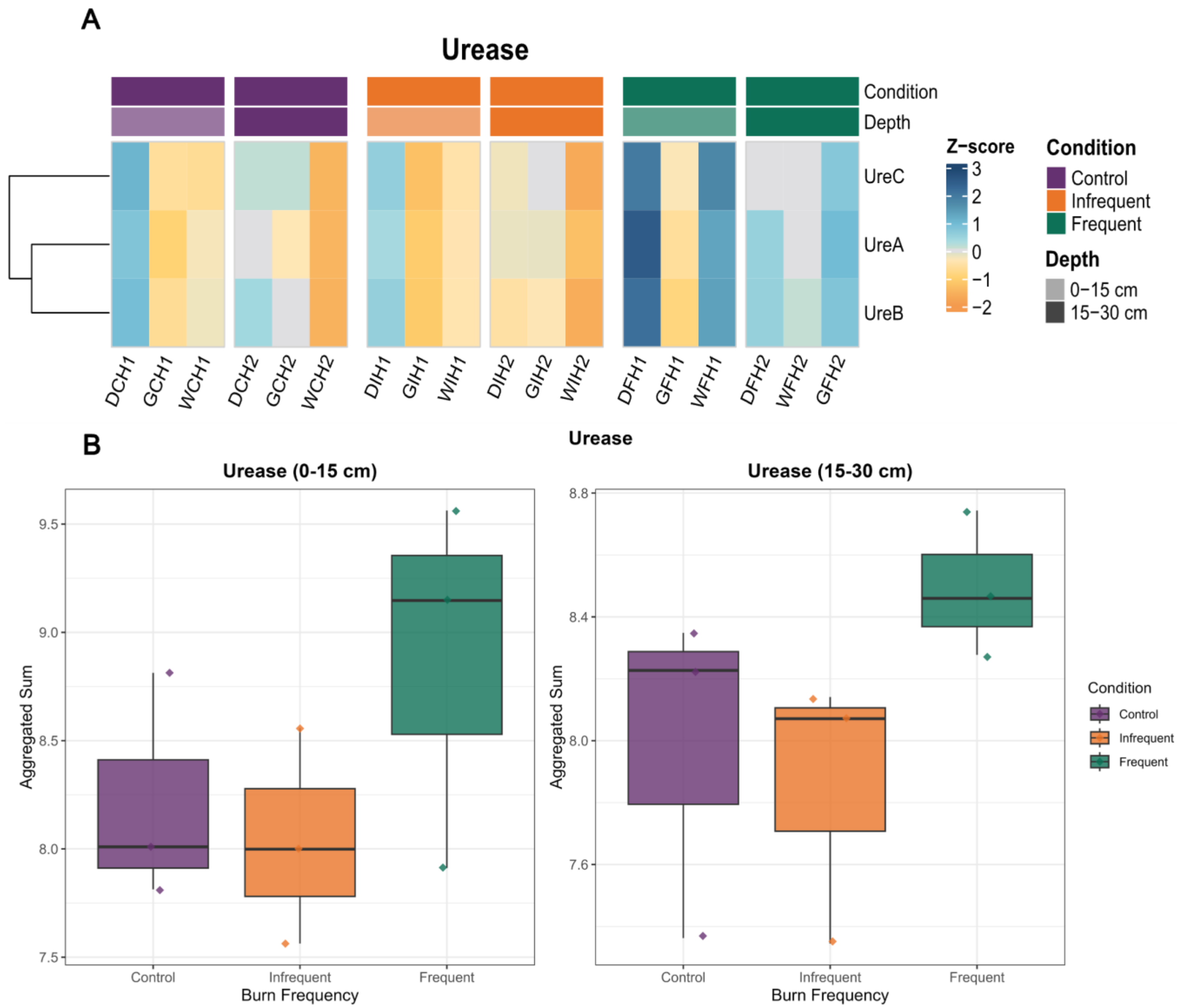

Figure S25. A) Normalized heatmap of gene present within the urease pathway ($NH_2CONH_2 \rightarrow NH_4^+$). Each row represents an individual gene and each column represents a sample, grouped by burn frequency treatment (Control, Infrequent, Frequent) and soil depth (0–15 cm, lighter shading; 15–30 cm, darker shading). Gene abundance values were normalized by Z-score across samples, such that each value reflects the number of standard deviations above or below the mean abundance for that gene across all samples. Blue indicates above-average abundance (positive Z-score), yellow-orange indicates below-average abundance (negative Z-score), and grey cells indicate an abundance of zero. Completely greyed-out rows indicate genes for which no data were available across any sample. **B)** box-and-whisker plot of all genes in Urease pathway from each site in both soil depth (0-15 cm) and (15-30 cm) respectively. Gene data were aggregated and clustered by pathway. ANOVA Tukey post hoc statisical signifance signified: * = *p <0.05, ** = p <0.01, *** = p <0.001*, no stars = no significance. Details on the genes grouped in each pathway are shown in **Table S4** while the pathways themselves are visualized in **Figure S2.**

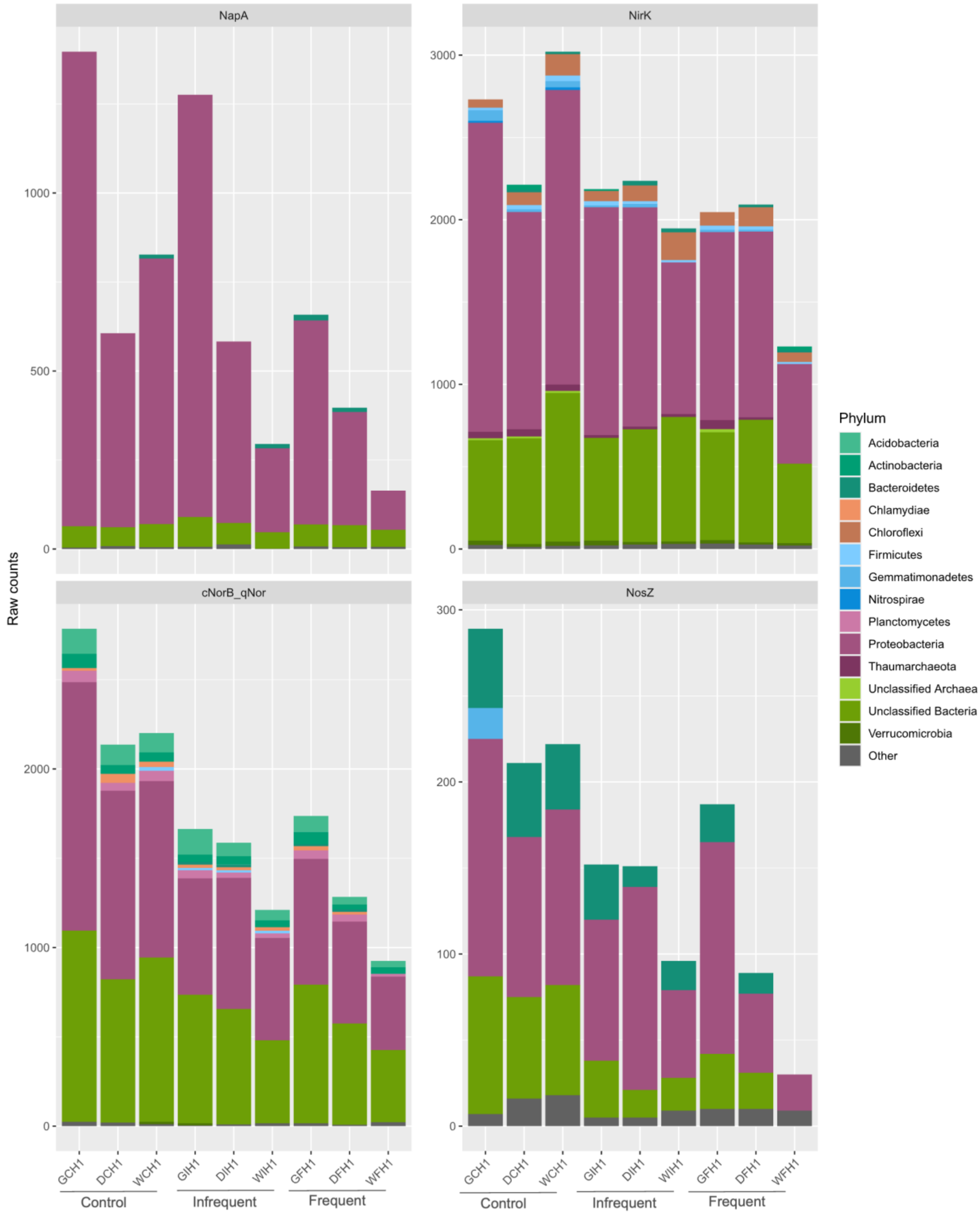

Figure S26: Raw count read of nitrogen cycling functional genes *napA* (Nitrate dissimilarity reduction pathway), *nirK* (Denitrification pathway), *qnor* (Denitrification pathway), and *nosZ* (Denitrification pathway) across all sites at soil depth 0-15 cm. Colors correlate to relevant phyla taxonomy, with prokaryotic raw count ≤10 grouped as "Other".

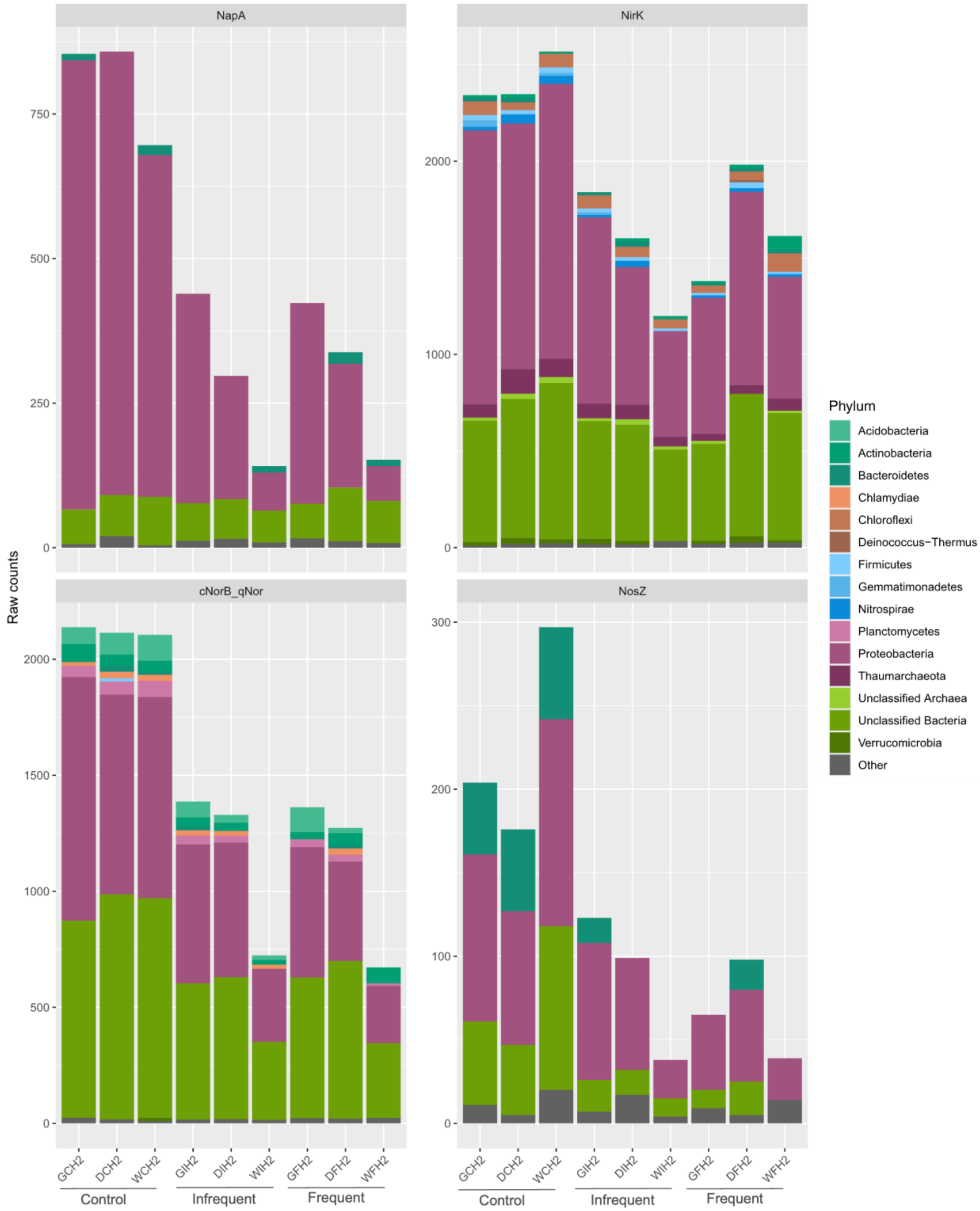

Figure S27: Raw count read of nitrogen cycling functional genes *napA* (Nitrate dissimilarity reduction pathway), *nirK* (Denitrification pathway), *norB* (Denitrification pathway), and *nosZ* (Denitrification pathway) across all sites at soil depth 15-30 cm. Colors correlate to relevant phyla taxonomy with prokaryotic raw count ≤10 grouped as "Other".

References for Supplemental Information

1. Barnes JK, Institute NYSBR, Museum NYS. 2003. Natural history of the Albany Pine Bush : Albany and Schenectady counties, New York, including a field guide and trail map. Albany, N.Y, New York State Museum, University of the State of New York, State Education Dept, c2003. https://www.biodiversitylibrary.org/item/239267.

2. Gifford NA. 2020. 2020 Hindsight: 28 Years of Restoring Fire's Role in an Urban Pine Barrens Preserve. Lake State Fire Science Consortium.

3. Lookingbill T, Brickle M, Engelhardt K. 2013. Evaluation of Albany Pine Bush. Evaluation.

4. Albany Pine Bush Preserve Commission. 2017. 2017 Managment Plan Update for The Abany Pine Bush Preserve. https://albanypinebush.org/fckimages/uploads/1525371460_2017%20Management%20Plan%20Update.pdf. Retrieved 25 April 2026.

5. Bried JT, Gifford NA. 2008. Albany Pine Bush Pine Barrens Viability Assessment.

6. Natural Resources Conservation Service, USDA. 2025. Web Soil Survey.

7. Tremblay M, Stager JC, St-Jacques J-M, Murphy S, Peros M, Carl BS. 2024. A 13,000-year history of vegetation and fire in a rare inland pine barrens: The Albany Pine Bush (Albany County, New York, USA). PLOS ONE 19:e0314101.

8. Wolfe SA, Franzi D, Carl B, Ferguson N, Woronko B, Chmielowska-Michalak D, Pantoja G, Schaarschmidt M, Lian OB, Gontz A, Gifford N. 2025. Origin and Late Pleistocene to early Holocene activity of the Albany Pine Bush dune field, New York, northeastern USA. Aeolian Res 74:100992.

9. Overbeek R, Olson R, Pusch GD, Olsen GJ, Davis JJ, Disz T, Edwards RA, Gerdes S, Parrello B, Shukla M, Vonstein V, Wattam AR, Xia F, Stevens R. 2014. The SEED and the Rapid Annotation of microbial genomes using Subsystems Technology (RAST). Nucleic Acids Res 42:D206–D214.

10. Kanehisa M, Goto S. 2000. KEGG: Kyoto Encyclopedia of Genes and Genomes. Nucleic Acids Res 28:27–30.

11. Buchfink B, Xie C, Huson DH. 2015. Fast and sensitive protein alignment using DIAMOND. Nat Methods 12:59–60.

12. Nayfach S, Pollard KS. 2015. Average genome size estimation improves comparative metagenomics and sheds light on the functional ecology of the human microbiome. Genome Biol 16:51.

13. Einsle O, Messerschmidt A, Stach P, Bourenkov GP, Bartunik HD, Huber R, Kroneck PMH. 1999. Structure of cytochrome c nitrite reductase. Nature 400:476–480.

14. Hussain H, Grove J, Griffiths L, Busby S, Cole J. 1994. A seven-gene operon essential for formate-dependent nitrite reduction to ammonia by enteric bacteria. Mol Microbiol 12:153–163.

15. Hird K, Campeciño JO, Hegg EL. 2025. From genes to function: regulation, maturation, and evolution of cytochrome c nitrite reductase in nitrate reduction to ammonium. Appl Environ Microbiol 91:e00292-25.

16. Rodrigues ML, Pereira IA, Archer M. 2008. The NrfH Cytochrome c Quinol DehydrogenaseHandbook of Metalloproteins. John Wiley & Sons, Ltd.

17. Simon J, Gross R, Einsle O, Kroneck PMH, Kröger A, Klimmek O. 2000. A NapC/NirT-type cytochrome c (NrfH) is the mediator between the quinone pool and the cytochrome c nitrite reductase of Wolinella succinogenes. Mol Microbiol 35:686–696.

18. Kern M, Mager AM, Simon J. 2007. Role of individual nap gene cluster products in NapC-independent nitrate respiration of Wolinella succinogenes. Microbiology 153:3739–3747.

19. Norton JM, Alzerreca JJ, Suwa Y, Klotz MG. 2002. Diversity of ammonia monooxygenase operon in autotrophic ammonia-oxidizing bacteria. Arch Microbiol 177:139–149.

20. Whittaker M, Bergmann D, Arciero D, Hooper AB. 2000. Electron transfer during the oxidation of ammonia by the chemolithotrophic bacterium *Nitrosomonas europaea*. Biochim Biophys Acta BBA - Bioenerg 1459:346–355.

21. Vossenberg J, Woebken D, Maalcke W, Wessels H, Dutilh B, Kartal B, Janssen-Megens E, Roeselers G, Yan J, Speth D, Gloerich J, Geerts W, Biezen E, Pluk W, Francoijs K-J, Russ L, Lam P, Malfatti S, Tringe S, Jetten M. 2012. The metagenome of the marine anammox bacterium 'Candidatus Scalindua profunda' illustrates the versatility of this globally important nitrogen cycle bacterium. Environ Microbiol 15.

22. Jiang Q, Cao L, Han Y, Li S, Zhao R, Zhang X, Ruff SE, Zhao Z, Peng J, Liao J, Zhu B, Wang M, Lin X, Dong X. 2025. Cold seeps are potential hotspots of deep-sea nitrogen loss driven by microorganisms across 21 phyla. Nat Commun 16.

23. Gerbl F, Weidler G, Wanek W, Erhardt A, Stan-lotter H. 2014. Thaumarchaeal ammonium oxidation and evidence for a nitrogen cycle in a subsurface radioactive thermal spring in the Austrian Central Alps. Front Microbiol 5:225.

24. Bricio C, Alvarez L, San Martin M, Schurig-Briccio LA, Gennis RB, Berenguer J. 2014. A Third Subunit in Ancestral Cytochrome c-Dependent Nitric Oxide Reductases. Appl Environ Microbiol 80:4871–4878.

25. Kobayashi H, Kawasaki K, Takeishi K, Noda H. 2011. Symbiont of the stink bug Plautia stali synthesizes rough-type lipopolysaccharide. Microbiol Res 167:48–54.

26. Chan DTC, Bernstein HC. 2024. Pangenomic landscapes shape performances of a synthetic genetic circuit across Stutzerimonas species. mSystems 9:e00849-24.

27. Braker G, Zhou J, Wu L, Devol AH, Tiedje JM. 2000. Nitrite Reductase Genes (nirK and nirS) as Functional Markers To Investigate Diversity of Denitrifying Bacteria in Pacific Northwest Marine Sediment Communities. Appl Environ Microbiol 66:2096–2104.

28. Intrator N, Ward BB. 2025. Aquatic copper-containing nitrite reductase gene (nirK) phylogeny and environmental distribution. Front Microbiol 16.

29. Alvarez L, Bricio C, José Gómez M, Berenguer J. 2011. Lateral Transfer of the Denitrification Pathway Genes among Thermus thermophilus Strains. Appl Environ Microbiol 77:1352–1358.

30. Adamczack J, Hoffmann M, Papke U, Haufschildt K, Nicke T, Bröring M, Sezer M, Weimar R, Kuhlmann U, Hildebrandt P, Layer G. 2014. NirN Protein from Pseudomonas aeruginosa is a Novel Electron-bifurcating Dehydrogenase Catalyzing the Last Step of Heme d1 Biosynthesis *. J Biol Chem 289:30753–30762.

31. Menestreau M, Milligan DA, Sennett LB, Bergaust L, Bakken LR, Rowley G, Kjos M, Shapleigh JP, Frostegård Å. 2026. Distinct Denitrification Phenotypes in Closely Related Bacteria: Clues to Understanding Variations in Nitrite Accumulation Among Stutzerimonas Strains. Environ Microbiol 28:e70275.

32. Ogawa K, Akagawa E, Yamane K, Sun ZW, LaCelle M, Zuber P, Nakano MM. 1995. The nasB operon and nasA gene are required for nitrate/nitrite assimilation in Bacillus subtilis. J Bacteriol 177:1409–1413.

33. Jepson BJN, Mohan S, Clarke TA, Gates AJ, Cole JA, Butler CS, Butt JN, Hemmings AM, Richardson DJ. 2007. Spectropotentiometric and Structural Analysis of the Periplasmic Nitrate Reductase from Escherichia coli*. J Biol Chem 282:6425–6437.

34. Cole J. 1996. Nitrate reduction to ammonia by enteric bacteria: redundancy, or a strategy for survival during oxygen starvation? FEMS Microbiol Lett 136:1–11.

35. Lin Y-C, Sekedat MD, Cornell WC, Silva GM, Okegbe C, Price-Whelan A, Vogel C, Dietrich LEP. 2018. Phenazines Regulate Nap-Dependent Denitrification in Pseudomonas aeruginosa Biofilms. J Bacteriol 200:10.1128/jb.00031-18.

36. Yang Z, Han Y, Ma Y, Chen Q, Zhan Y, Lu W, Cai L, Hou M, Chen S, Yan Y, Lin M. 2018. Global investigation of an engineered nitrogen-fixing Escherichia coli strain reveals regulatory coupling between host and heterologous nitrogen-fixation genes. Sci Rep 8:10928.

37. Tu Q, Lin L, Cheng L, Deng Y, He Z. 2019. NCycDB: a curated integrative database for fast and accurate metagenomic profiling of nitrogen cycling genes. Bioinformatics 35:1040–1048.

38. NasJ - Fama. https://iseq.lbl.gov/fama/reference/nitrogen/NasJ/. Retrieved 25 May 2026.

39. Flores E, Frías JE, Rubio LM, Herrero A. 2005. Photosynthetic nitrate assimilation in cyanobacteria. Photosynth Res 83:117–133.

40. Peng Y, He T, Zhou Q, Yin M, Jin C. 2026. The functional analysis of NirBD in microorganisms and the efficacies of NirBD on the soil nitrogen storage and N2O emission. Synth Syst Biotechnol 12:364–373.

41. Harborne NR, Griffiths L, Busby SJW, Cole JA. 1992. Transcriptional control, translation and function of the products of the five open reading frames of the Escherichia coli nir operon. Mol Microbiol 6:2805–2813.

42. Siciliano SD, Roy R. 2000. Reduction in denitrification activity in field soils exposed to long term contamination by 2,4,6-trinitrotoluene (TNT). FEMS Microbiol Ecol 32:61–68.

43. Yang J, Xie X, Wang X, Dixon R, Wang Y-P. 2014. Reconstruction and minimal gene requirements for the alternative iron-only nitrogenase in Escherichia coli. Proc Natl Acad Sci 111:E3718–E3725.

44. Jiang X, Coroian D, Barahona E, Echavarri-Erasun C, Castellanos-Rueda R, Eseverri Á, Aznar-Moreno JA, Burén S, Rubio LM. 2022. Functional Nitrogenase Cofactor Maturase NifB in Mitochondria and Chloroplasts of Nicotiana benthamiana. mBio 13.

45. Fani R, Gallo R, Liò P. 2000. Molecular Evolution of Nitrogen Fixation: The Evolutionary History of the nifD, nifK, nifE, and nifN Genes. J Mol Evol 51:1–11.

46. Joerger RD, Jacobson MR, Premakumar R, Wolfinger ED, Bishop PE. 1989. Nucleotide sequence and mutational analysis of the structural genes (anfHDGK) for the second alternative nitrogenase from Azotobacter vinelandii. J Bacteriol 171:1075–1086.

47. Ininbergs K, Bay G, Rasmussen U, Wardle DA, Nilsson M-C. 2011. Composition and diversity of nifH genes of nitrogen-fixing cyanobacteria associated with boreal forest feather mosses. New Phytol 192:507–517.

48. Cruz-Ramos H, Glaser P, Wray LV, Fisher SH. 1997. The Bacillus subtilis ureABC operon. J Bacteriol 179:3371–3373.

49. Feng T, Miao P, Zheng B, Li W, Sukkaew E, Chen Y, Wang J, Peng Y, Zheng W, Zhai B. 2025. Soil urease activity is mediated more by ureC microbial diversity in wet regions than dry regions: Key roles of generalist-abundant and -rare species. Funct Ecol 39:2015–2028.
